# Supplementary material for: Facial soft tissue thickness in forensic facial reconstruction: Impact of regional differences in Brazil
Source: PLoS One. 2022 Jul 15;17(7):e0270980. doi: 10.1371/journal.pone.0270980 (PMC9286276; doi:10.1371/journal.pone.0270980)
Supplement: S1 Table — (PDF) [file pone.0270980.s001.pdf]

S1 Table. ICC Intra- and Interexaminer

Landmark	ICC intra-examiner	ICC inter-examiner
Supraglabellare	0.993	0.961
Glabella	0.99	0.967
Nasion	0.993	0.881
Rhinion	0.968	0.715
Mid-Philtrum	0.988	0.973
Prosthion	0.982	0.973
Infradentale	0.995	0.947
Supramentale	0.95	0.641
Pogonion	0.996	0.994
Menton	0.993	0.979
Frontal Eminence R	0.993	0.979
Frontal Eminence L	0.987	0.958
Mid-supraorbital R	0.992	0.968
Mid-supraorbital L	0.995	0.951
Mid-infraorbital R	0.995	0.980
Mid-infraorbital L	0.991	0.979
Malar R	0.986	0.890
Malar L	0.978	0.837
Lateral Orbital R	0.985	0.986
Lateral Orbital L	0.991	0.988
Zygion R	0.991	0.949
Zygion L	0.995	0.980
Supraglenoid R	0.99	0.980
Supraglenoid L	0.996	0.879
Gonion R	0.998	0.989
Gonion L	0.998	0.983
Ectomolare^2 R	0.978	0.943
Ectomolare^2 L	0.996	0.931
Occlusal Line R	0.998	0.995
Occlusal Line L	0.999	0.995
Ectomolare$_2$ R	0.996	0.945
Ectomolare$_2$ L	0.995	0.941
All landmarks	0.999	0.995
