# Supplementary material for: Facial soft tissue thickness in forensic facial reconstruction: Impact of regional differences in Brazil
Source: PLoS One. 2022 Jul 15;17(7):e0270980. doi: 10.1371/journal.pone.0270980 (PMC9286276; doi:10.1371/journal.pone.0270980)
Supplement: S2 Table — (PDF) [file pone.0270980.s002.pdf]

S2 Table. FSTT Means in Age Groups in Both Sexes

	Female			Male		
**Landmark**	**18 to 30y**	**31 to 40y**	**41 an older**	**18 to 30y**	**31 to 40y**	**41 an older**
Supraglabellare	4.68	4.54	4.14	5.35	4.90	6.09
Glabella	4.93	4.63	5.18	5.88	5.94	6.02
Nasion	6.40	5.93	6.26	8.15	8.05	8.16
Rhinion	1.76	1.53	1.79	2.23	2.21	2.34
Mid-Philtrum	13.76	13.41	12.06	16.18	14.87	15.94
Prosthion	10.85	10.32	8.89	13.77	13.02	12.91
Infradentale	9.09	10.04	10.39	11.16	11.96	12.87
Supramentale	11.42	11.91	12.32	12.76	12.52	13.47
Pogonion	9.35	9.44	10.54	11.10	10.79	12.41
Menton	6.83	6.77	7.32	9.42	7.89	9.43
Frontal Eminence R	4.38	3.86	3.83	4.64	4.64	5.61
Frontal Eminence L	4.66	4.08	3.61	4.55	4.91	5.36
Mid-supraorbital R	6.42	5.70	7.12	8.64	8.33	8.76
Mid-supraorbital L	6.38	5.51	6.96	8.74	8.54	8.86
Mid-infraorbital R	5.29	5.30	5.24	5.36	5.29	6.80
Mid-infraorbital L	5.65	5.33	5.36	5.51	5.40	6.89
Malar R	21.39	21.12	21.65	22.47	22.46	23.65
Malar L	21.91	20.77	22.14	22.48	22.54	23.86
Lateral Orbital R	9.42	8.69	8.97	8.31	7.96	8.54
Lateral Orbital L	9.65	8.61	9.05	8.20	8.19	8.68
Zygion R	8.09	7.66	7.70	9.28	9.32	9.14
Zygion L	8.18	7.35	7.60	8.87	9.36	9.17
Supraglenoid R	10.32	10.82	10.92	12.34	12.80	13.34
Supraglenoid L	10.58	10.68	10.86	12.02	13.06	13.42
Gonion R	12.42	12.65	13.43	16.17	18.57	20.80
Gonion L	12.69	12.69	13.44	15.65	18.34	20.09
$Ectomolare^2$ R	28.11	27.12	25.76	30.53	29.45	30.90
$Ectomolare^2$ L	28.67	26.97	25.82	30.06	29.60	30.84
Occlusal Line R	20.63	19.90	20.16	24.32	23.94	25.67
Occlusal Line L	20.92	20.12	20.09	23.99	23.85	25.01
$Ectomolare_2$ R	25.20	24.28	24.90	27.54	28.21	29.14
$Ectomolare_2$ L	25.64	24.34	24.24	27.45	28.73	29.40
