# Supplementary material for: Facial soft tissue thickness in forensic facial reconstruction: Impact of regional differences in Brazil
Source: PLoS One. 2022 Jul 15;17(7):e0270980. doi: 10.1371/journal.pone.0270980 (PMC9286276; doi:10.1371/journal.pone.0270980)
Supplement: Attachment — Submitted filename: PONE-D-22-02392_R1.pdf [file pone.0270980.s004.pdf]

PLOS ONE

Facial Soft Tissue thickness in Forensic Facial Reconstruction: Impact of regional differences in Brazil

--Manuscript Draft--

**Manuscript Number:**	PONE-D-22-02392R1
**Article Type:**	Research Article
**Full Title:**	Facial Soft Tissue thickness in Forensic Facial Reconstruction: Impact of regional differences in Brazil
**Short Title:**	Regional differences on facial thickness
**Corresponding Author:**	Deisy Moritsugui<br>University of Sao Paulo: Universidade de Sao Paulo<br>São Paulo, BRAZIL
**Keywords:**	forensic facial reconstruction; forensic facial approximation; human identification; cone beam computed tomography; forensic anthropology.
**Abstract:**	Abstract<br>Forensic facial reconstruction aims to assemble and provide the appearance of a face over a skull with the intent of allowing the recognition of that individual, expecting the antemortem information to be presented, and, therefore, making possible the application of primary identification methods. The scientific literature applies reference tables regarding the facial soft tissue thickness (FSTT) in a range of different geographic regions. However, the consensus on its importance or on how to use specific population data related to FSTT is not unanimous. Brazil is formed by geographic regions with diverse environmental characteristics and populations, which are reflected in facial features. This paper aimed to measure and compare FSTT of distinct Brazilian samples to ascertain the need for specific data sets for different regions. A specific protocol for cone beam computed tomography was used to standardize measurement, and it was applied in a sample of 101 exams. The FSTT measurements of a Brazilian population from the Midwest Region was compared to a previous sample from Southeast, which was collected using the same protocol. High compatibility was observed when comparing the averages of FSTT among samples of these two different geographic regions. Regarding age groups, notable differences on the medium and inferior face were observed in females. Minor variances found are unlikely to affect the practice of forensic facial reconstruction. Facial features, such as eyes, lips, nose, and skin may also be relevant in the differentiation of people from these 2 areas in Brazil. Therefore, concerning the Southeast and Midwest Brazilian regions, the need to apply different data sets is unnecessary.
**Order of Authors:**	Deisy Moritsugui
	Flavia Vanessa Greb Fugiwara
	Flavia Nicolle Stefani Vassallo
	Luiz Eugênio Nigro Mazzilli, PhD
	Thiago Leite Beaini, PhD
	Rodolfo Francisco Haltenhoff Melani, PhD
**Opposed Reviewers:**	
**Response to Reviewers:**	The authors would like to thank the reviewers for their valuable comments and excellent suggestions to improve the quality of our manuscript.<br>The authors have revised all points brought by the reviewers. We sincerely hope that the information presented in this manuscript provides some answers and opens novel questions to advance future research.
**Additional Information:**	
**Question**	**Response**
**Financial Disclosure**	The author(s) received no specific funding for this work.

Powered by Editorial Manager® and ProduXion Manager® from Aries Systems Corporation

Enter a financial disclosure statement that describes the sources of funding for the work included in this submission. Review the submission guidelines for detailed requirements. View published research articles from *PLOS ONE* for specific examples.<br><br>This statement is required for submission and **will appear in the published article** if the submission is accepted. Please make sure it is accurate.<br><br>**Unfunded studies**<br>Enter: *The author(s) received no specific funding for this work.*<br><br>**Funded studies**<br>Enter a statement with the following details:<br>• Initials of the authors who received each award<br>• Grant numbers awarded to each author<br>• The full name of each funder<br>• URL of each funder website<br>• Did the sponsors or funders play any role in the study design, data collection and analysis, decision to publish, or preparation of the manuscript?<br>• **NO** - Include this sentence at the end of your statement: *The funders had no role in study design, data collection and analysis, decision to publish, or preparation of the manuscript.*<br>• **YES** - Specify the role(s) played.<br><br>* typeset	
**Competing Interests**<br><br>Use the instructions below to enter a competing interest statement for this submission. On behalf of all authors, disclose any competing interests that could be perceived to bias this work—acknowledging all financial support and any other relevant financial or non-financial competing interests.<br><br>This statement is required for submission and **will appear in the published article** if	The authors have declared that no competing interests exist.

the submission is accepted. Please make sure it is accurate and that any funding sources listed in your Funding Information later in the submission form are also declared in your Financial Disclosure statement.<br><br>View published research articles from *PLOS ONE* for specific examples.<br><br>**NO authors have competing interests**<br><br>Enter: *The authors have declared that no competing interests exist.*<br><br>**Authors with competing interests**<br><br>Enter competing interest details beginning with this statement:<br><br>*I have read the journal's policy and the authors of this manuscript have the following competing interests: [insert competing interests here]*<br><br>* typeset	
**Ethics Statement**<br><br>Enter an ethics statement for this submission. This statement is required if the study involved:<br><br>• Human participants<br>• Human specimens or tissue<br>• Vertebrate animals or cephalopods<br>• Vertebrate embryos or tissues<br>• Field research<br><br>Write "N/A" if the submission does not require an ethics statement.<br><br>General guidance is provided below. Consult the submission guidelines for detailed instructions. **Make sure that all information entered here is included in the Methods section of the manuscript.**	This study was approved by the Committee of Ethics in Research of the School of Dentistry of University of São Paulo (CAAE 33433320.8.0000.007 and 33433320.8.0000.0075). The form consent was not obtained since we used a database and we analyzed it anonymously

**Format for specific study types**<br><br>**Human Subject Research (involving human participants and/or tissue)**<br>• Give the name of the institutional review board or ethics committee that approved the study<br>• Include the approval number and/or a statement indicating approval of this research<br>• Indicate the form of consent obtained (written/oral) or the reason that consent was not obtained (e.g. the data were analyzed anonymously)<br><br>**Animal Research (involving vertebrate animals, embryos or tissues)**<br>• Provide the name of the Institutional Animal Care and Use Committee (IACUC) or other relevant ethics board that reviewed the study protocol, and indicate whether they approved this research or granted a formal waiver of ethical approval<br>• Include an approval number if one was obtained<br>• If the study involved *non-human primates*, add *additional details* about animal welfare and steps taken to ameliorate suffering<br>• If anesthesia, euthanasia, or any kind of animal sacrifice is part of the study, include briefly which substances and/or methods were applied<br><br>**Field Research**<br><br>Include the following details if this study involves the collection of plant, animal, or other materials from a natural setting:<br>• Field permit number<br>• Name of the institution or relevant body that granted permission	
**Data Availability**<br><br>Authors are required to make all data underlying the findings described fully available, without restriction, and from the time of publication. PLOS allows rare exceptions to address legal and ethical concerns. See the PLOS Data Policy and FAQ for detailed information.	Yes - all data are fully available without restriction

A Data Availability Statement describing where the data can be found is required at submission. Your answers to this question constitute the Data Availability Statement and **will be published in the article**, if accepted.<br><br>**Important:** Stating 'data available on request from the author' is not sufficient. If your data are only available upon request, select 'No' for the first question and explain your exceptional situation in the text box.<br><br>Do the authors confirm that all data underlying the findings described in their manuscript are fully available without restriction?	
**Describe where the data may be found in full sentences. If you are copying our sample text, replace any instances of XXX with the appropriate details.**<br><br>• If the data are **held or will be held in a public repository**, include URLs, accession numbers or DOIs. If this information will only be available after acceptance, indicate this by ticking the box below. For example: *All XXX files are available from the XXX database (accession number(s) XXX, XXX.).*<br>• If the data are all contained **within the manuscript and/or Supporting Information files**, enter the following: *All relevant data are within the manuscript and its Supporting Information files.*<br>• If neither of these applies but you are able to provide **details of access elsewhere**, with or without limitations, please do so. For example:<br><br>*Data cannot be shared publicly because of [XXX]. Data are available from the XXX Institutional Data Access / Ethics Committee (contact via XXX) for researchers who meet the criteria for access to confidential data.*<br><br>*The data underlying the results presented in the study are available from (include the name of the third party*	All relevant data are within the manuscript file.

and contact information or URL).<br>• This text is appropriate if the data are owned by a third party and authors do not have permission to share the data.<br><br>* typeset	
Additional data availability information:	

Cover letter

University of São Paulo
School of Dentistry - Department of Social Dentistry
Av. Prof. Lineu Prestes, 2227 – Butantã, São Paulo – SP - Brazil

Dr. Dong Hoon Shin
Editor-in-Chief
Plos One

January 25th, 2022

Dear Dr. Shin,

We wish to submit an original research article entitled "Facial Soft Tissue thickness in Forensic Facial Reconstruction: Impact of regional differences and age effect" for consideration by Plos One.

In this paper, we evaluated two variables related to facial thickness which is essencial for Facial Reconstruction. The first concerns to distinct geographic regions and the second is related to facial aging process. We believe that this manuscript is appropriate for publication by Plos One because it can contribute for facial soft tissue thickness data bases worldwide and consequently accurate outcomes can be achieved. Furthermore, the co-authors, Thiago Beaini and Rodolfo Melani published, in recent years, two papers in Plos One related to the same line of research (doi.org/10.1371/journal.pone.0196770 , DOI:10.1371/journal.pone.0162732).

There has been a continuing investigation on facial soft tissue thickness and the role of specific data set according to sex, age, population affinity or geographic region, thereby improving Forensic Facial Reconstruction as an auxiliary identification method on forensic cases. Our results confirm that discrepancies related to different geographic regions have little practical meaning for the recognition procedure. It was verified that facial aging process modifies significantly facial thicknesses.

This study was approved by the Committee of Ethics in Research of the School of Dentistry of University of São Paulo (protocol 4.191.247). We confirm that the paper was approved by all authors and that we do not have any conflict of interest to disclose.

We confirm that this work is original and has not been published elsewhere, nor is it currently under consideration for publication elsewhere.

Please address all correspondence concerning this manuscript to me at dsmoritsugui@usp.br

Thank you for your consideration.

Sincerely,

Deisy Satie Moritsugui

Manuscript Click here to access/download;Manuscript;Manuscript.docx

Facial soft tissue thickness in forensic facial reconstruction: impact of regional differences in Brazil

Deisy Satie Moritsugui[1¶*], Flavia Vanessa Greb Fugiwara[1&], Flávia Nicolle Stefani Vassallo[1&], Luiz Eugênio Nigro Mazzilli[1¶], Thiago Leite Beaini[2¶], Rodolfo Francisco Haltenhoff Melani[1¶]

[1] Laboratory of Forensic Anthropology and Odontology (OFLAB), Department of Social Dentistry, School of Dentistry, University of São Paulo, São Paulo, São Paulo, Brazil

[2]Department of Preventive and Social Dentistry, School of Dentistry, Federal University of Uberlândia, Uberlândia, Minas Gerais, Brazil

*Corresponding author

E-mail: dsmoritsugui@usp.br (DSM)

[¶] These authors contributed equally to this work.

[&] These authors also contributed equally to this work.

Abstract

Forensic facial reconstruction aims to assemble and provide the appearance of a face over a skull ~~with the intent of allowing the~~ recognition of that individual, ~~expecting the antemortem information to be presented, and, therefore,~~ making possible the application of primary identification methods. The scientific literature ~~applies reference tables regarding the~~ facial soft tissue thickness (FSTT) ~~in~~ a range of different geographic regions. However, the consensus on its importance or on how to use specific population data related to FSTT is not unanimous. Brazil is formed by geographic regions with diverse ~~environmental characteristics and~~ populations, which are reflected in facial features. This paper aimed to measure and compare FSTT of distinct Brazilian samples to ascertain the need for specific data sets for different regions. A specific protocol for cone beam computed tomography was used to standardize measurement, and it was applied in a sample of 101 ~~exams~~. The FSTT measurements of a Brazilian population from the Midwest Region was compared to a previous sample from Southeast, which was collected using the same protocol. High compatibility was observed when comparing the averages of FSTT among samples of these two different geographic regions. Regarding age groups, notable differences on the medium and inferior face were observed in females. Minor variances found are unlikely to affect the practice of forensic

facial reconstruction. Facial features, such as eyes, lips, nose, and skin may also be relevant in the differentiation of people from these areas in Brazil. Therefore, concerning the Southeast and Midwest Brazilian regions, the need to apply different data sets is unnecessary.

Keywords: forensic facial reconstruction, forensic facial approximation, human identification, cone beam computed tomography, forensic anthropology.

Introduction

~~The~~ forensic facial reconstruction (FFR) has an important role in recognizing individuals who are unable to be identified by other methods. Advanced postmortem decomposition process or the lack of antemortem information are its main indication for forensic purposes [1,2]. The technique is based on the assembling of an approximated soft tissue topography over the unknown skull, with the intent of reconstructing its characteristics to be as similar as possible to the individual at the time of ~~his or her~~ death. Once the reconstruction is finished, a public campaign is issued, aiming to illicit a response from the public as to whom the remains might belong. Following this, a formal identification can then take place [1,3,4].

An approximated face is achievable by using various techniques. The 2D representation is based on a picture of the skull, whereas the 3D

representation can be divided into manual or digital methods. The manual method involves direct modelling and sculpture, applying clay or Plasticine over the skull or a model of the skull [1]. More recently, a computerized face sculpture can be generated by using specialized modeling software, and different user interfaces can adapt the procedures to resemble manual modeling. Tablets and the assistance of haptic devices may be used in the 3D context to add soft tissues to a model of the skull [5]. However, independently of the method used, it is imperative to establish parameters and guidance such as the facial soft tissue thickness (FSTT), that serves as reference over specific craniometric landmarks [6], Those FSTT measurements are usually populational-based averages, but from this general information it is often expected to deliver a precise and reliable assistance during FFR [7].

FSTT charts in the scientific literature cover many craniometric landmarks, allowing it to be organized by sex, age, ancestry, BMI, among other facial features considered to be significant in an FFR. Many aspects of the method that seem to be related to the success rate or to the probability of recognition of the reconstructed faces. Studies have indicated that FSTT data are particularly influenced by the population groups under study [8–12], but the debate on the subject has far from achieved a consensus. Therefore, the expert should consider using of FSTT based on the population to which the

body can be correlated by previous anthropological exams because specific FSTT charts could produce better results. However, in those studies, it is remarkable that many describe different methodologies, including the original measurement protocols, the source and type of imaging exam and the relation of regional charts, and the success of the FFR has not yet been established. ~~Hence, some state evidence to justify the need for applying different population data to FFR is insufficient [13], but the doubt still raises questions in situations such as the reality of countries such as Brazil.~~

The Brazilian territory has continental proportions, and it is divided into geographical regions (north, northeast, southeast, south, and Midwest), each one with its diverse environments and populations.

To understand the local context better, it is important to remember that the original population was based on several Indigenous Brazilian with different anthropological features among the groups found in the territory. The European colonization favored the concept of a mixed ancestry population, mostly influenced by the presence of Portuguese, Dutch and French. After the world wars of the 20th century, the presence of immigrants from Japan, Eastern Europe, the Middle East, Italy and Germany became important [14]. Their distribution was not even among the regions and, therefore, it is clear that each segment of Brazil has its own physical features that represent the dominant population groups, with different characteristics

that can be traced to one’s origin [15]. The increasing and continuous lack of ancestral uniformity, as it is observed in Brazil, raises the question considering if the usage of a single STT chart is capable to approximate one’s morphological facial features, in this great individual variety context [16].

This study considers that the skull-facial morphology is determined by hereditary features and influenced by the environment [11], affecting the skull and the face. The combination of these anatomical differences makes the individual unique, leading to a wide variation of facial profiles across the country [17]. I[illegible]ustifies deeper investigation and the collection of data from different regions. With the intent of verifying the need for various FSTT charts on reconstruction from each geographic region, this research aimed to measure the FSTT in a Brazilian sample from the Midwest region and to compare to a previous sample from the Southeast region [18] using the same cone-beam computed tomography (CBCT) measuring protocol.

Materials and methods

The sample was composed of 101 CBCT exams of Brazilian subjects from the Midwest region, available in a previously established anonymous data bank in Digital Imaging Communication in Medicine (DICOM) format. Table 1 shows sample distribution.

Table 1 – Sample distribution by sex and age group

Sex<br>Age group	Male	Female	Total
18 to 30 years	18	22	40
31 to 40 years	13	14	27
41 and older	14	20	34
**Total**	45	56	101

The exams were acquired from records of clinical diagnosis, and, therefore, no participant was exposed to ionizing radiation for this research. The image protocol had a 23 cm x 17 cm FOV field of view, allowing the visualization of all included anatomical structures and landmarks from the supraglabellare to menton in the vertical axis and posterior to the supraglenoid structure. The images were obtained with an i-CAT Next Generation tomograph with 17.8 second exposition and voxel of 0.3 mm, and they were exported on Digital Imaging and Communication in Medicine (DICOM) format. CBCT scans allow image acquisitions with the individual in a sitting or standing position. Despite containing stabilizing devices for the head, such as the cephalostat and the chin support, these were not used in our sample, ~~which avoided~~ the effects of compression of facial soft tissues.

The research was approved by the Committee of Ethics in Research of the School of Dentistry of University of São Paulo (CAAE 33433320.8.0000.007 and 33433320.8.0000.0075). The inclusion criteria were established by observing the facial integrity of the research subjects.

Therefore, it included the exams of adult patients (18 and older) with required presence of certain structures: the central incisors and second molars (including both natural and prosthetically rehabilitated elements), no visible facial syndromes (hard or soft tissue), with no severe asymmetry and that had not been submitted to orthognathic surgery. Furthermore, the research included exams of both male and female patients in balanced groups. Thus, the exclusion criteria rejected exams that did not fit all the inclusion criteria or had quality issues that could prejudice the measuring of soft tissues.

The selection and measurements were performed with the assistance of the Horos software (version 3.3.6, 64 bit, Horos Project, Purview, Annapolis, USA) installed on an iMac computer (macOS Catalina – version 10.25.2, a 3,4 GHz Intel Core i7 Quad-Core processor, 16 GB RAM) and the 3D reconstruction tool 3D volume rendering through the skin and bone filters. Horos software is an open access version of OsiriX (DICOM viewer software) and has demonstrated to be a good alternative for craniofacial anthropological analyses, both in hard and soft tissues [19].

The exams were divided by ~~individuals'~~ sex into 3 age groups (from 18 to 30 years old, from 31 to 40 years old, 41 and older) following the original research group divisions [18], whose control sample, from

Southeast Brazilian region, was composed of 100 exams equally distributed among the groups.

The protocol established by Beaini et al. [18] was used to create a standardized measurements of FSTT in CBCT. In this guide, the skull images were positioned with the Frankfurt pl[illegible] parallel to the horizontal axis to avoid the influence of misplacement on multiplanar view. The measurements of FSTT were executed in the same craniometric landmarks studied by Rhine and Campbell [20]. Thrirty-two craniometric landmarks were measured, 1[illegible]ocated on the midline and 11[illegible]ilateral landmarks. Figs 1-3 present the craniometric landmarks and measurement direction, whereas Table 2 describes each landmark and the measurement directions according to the applied protocol.

Fig 1. Craniometric Landmarks Measured

Fig 2. Sagittal Landmarks Measurements

Fig 3. Bilateral Landmarks Measurements

Table 2 – Craniometric Landmarks, Descriptions, and Measurement Direction

	Landmark	Description	Measurement direction
	**Median points**		

1	**Supraglabellare**	Deepest part of the supraglabella fossa in the median plane	Perpendicular to the bone surface
2	**Glabella**	Most projecting median point on lower edge of the frontal bone	Perpendicular to the bone surface
3	**Nasion**	Intersection of the nasofrontal sutures in the median plane	Nasal soft tissue fold
4	**Rhinion**	Most rostral point on the internasal suture	Perpendicular to the bone surface
5	**Mid-Philtrum**	Deepest point of the maxillary alveolar bone	Mid-Philtrum or subnasale
6	**Prosthion**	Median point between the central incisors on the anterior margin of the maxillary alveolar rim	Upper vermilion border
7	**Infradentale**	Median point at the superior tip of the septum between the mandibular central incisors	Lower vermilion border
8	**Supramentale**	Deepest median point in the groove superior to the mental eminence	Chin fold
9	**Pogonion**	Most anterior median point on the mental eminence of the mandible	Perpendicular to the bone surface
10	**Menton**	Most inferior median point of the mental symphysis	Vertical direction or to neck fold
	**Bilateral Points**		
11	**Frontal Eminence**	Most projecting point of the frontal bone, at a line that vertically bisects the orbit	Perpendicular to the bone surface
12	**Mid-supraorbital**	Point on the anterior aspect of the superior orbital rim, at a line that vertically bisects the orbit	Orthoradial direction
13	**Mid-infraorbital**	Point on the anterior aspect of the inferior orbital rim, at a line that vertically bisects the orbit	Orthoradial direction
14	**Malar**	Intersection of the maxillary alveolar process and zygomatic process, at a line that vertically bisects the orbit	Perpendicular to the bone surface
15	**Lateral Orbital**	On the center of the zygomatic bone, at a line that vertically crosses the lateral orbital margin	Orthoradial direction
16	**Zygion**	The most lateral point on the zygomatic arch	Orthoradial direction
17	**Supraglenoid**	Most rostral point on the superior rim of the glenoid cavity	Orthoradial direction
18	**Gonion**	Point on the rounded margin of the angle of the mandible, bisecting two lines one following vertical margin of ramus and one following horizontal margin of the corpus of mandible	Perpendicular to the bone surface

**19**	**Ectomolare^2**	Most lateral point on the buccal alveolar margin, at the center of the superior second molar position	Orthoradial direction
**20**	**Occlusal Line**	Most anterior point on the mandibular ramus, where the occlusal line meets de mandible	Orthoradial direction
**21**	**Ectomolare$_2$**	Most lateral point on the buccal alveolar margin, at the center of the inferior second molar position	Orthoradial direction

The DICOM files from CBCT were imported into the Horos software, according to the protocol by Beaini et al. [18], a window level of 500 e window width of 3500 were applied to all exams to favor the visualization of both hard and soft tissue limits. The acquired volume was reconstructed on a 3D MPR window (multiplanar reconstruction) and visualized with the aid of a MIP tool (maximum intensity projection) to perform the volume repositioning to the Frankfurt ~~plane reference~~, as previously mentioned.

An initial calibration was made in the form of inter- and intraobserver tests. The measurements for this research were collected only with a high concordance between observers. The evaluation and adjustment of inter and intra-examiners were achieved by testing the 32 landmarks in 15 exams (15% of the sample) and testing the intraclass correlation coefficient (ICC) for each point. Regarding intra-examiner assessment, the main examiner measured the 15 exams twice, considering a 1 week time interval

between the measurements. The main examiner's previous training of the method ~~aimed to agree with~~ the original examiner ~~of the compared research.~~

The data were organized in a spreadsheet, using the software Microsoft Excel, and statistical analysis was performed with aid of the SPSS statistic pack, establishing a reliability level of 95%.

Using the Shapiro-Wilk test, the normality distribution sample was checked for every landmark, with $P < 0.05$, which implies alteration of normality. In the nonnormal distribution landmarks, the bootstrap method was used (1000 resampling – 95% CI BCa) to obtain reliable average values.

To search for sex related differences, the *t*-test was applied on landmarks with normal distribution, whereas the Mann-Whitney test was used with nonnormal distribution data. The variance analysis from a one-way ANOVA was applied to investigate any differences among the average FSTT, by landmark, on the three age groups (18 to 30 years, 31 to 40 years and 41 and older).

Concerning the regional differences, the research data were compared to the raw data of the Southeast region [18] to which full access was granted. A *t*-test was applied on the comparison of FSTT averages among the Southeastern [18] and Midwestern (this study) regions, for both sexes.

Results

The concordance of the measurements verified by the ICC according to each landmark measurement revealed intra- and interexaminer agreement above 95%, demonstrating reliability among the measurements. Fig 4 presents the individual ICC records.

Fig 4. Concordance Level for Each Landmark in Intra- and Interobserver Analysis

For male subjects, the Shapiro-Wilk test revealed that, from the 32 analyzed landmarks, 2 presented nonnormal distribution (lateral orbital and ectomolare^2). Female subjects presented nonnormal distribution at the landmarks rhinion, mid-infraorbital, zygion and gonion. In nonnormal distribution landmarks, the bootstrap method (1000 resamples – 95% CI BCa) was used to obtain reliable mean values. Such statistical procedure assigns consistent results, through the process of random resampling, because the process tends to correct normality deviations of the sample [21].

Table 3 exhibits the FSTT averages as the main result of this research. It has been divided by sex with SD, SE and *P* values, corresponding to each test, verifying statistically significant differences between the female and male averages, after bootstrapping procedure.

Table 3 – Mean FSTT (Millimeters and Percentage), SD, and SE Divided by Sex

	Female			Male					
	Mean	SD	SE	Mean	SD	SE	Mean difference (mm) **	Mean difference (%) ***	*P* value
Supraglabella	**4.5**	0.97	0.14	**5.5**	1.14	0.23	-1	18.1	0.00* T
Glabella	**4.9**	0.77	0.1	**5.9**	0.95	0.14	-1	16.9	0.00* T
Nasion	**6.2**	0.97	0.13	**8.1**	1.24	0.18	-1.9	23.4	0.00* T
Rhinion	**1.7**	0.43	0.06	**2.3**	0.58	0.09	-0.5	26.0	0.00* M
Mid-Philtrum	**13.1**	1.82	0.24	**15.7**	2.22	0.33	-2.7	16.5	0.00* T
Prosthion	**10**	1.58	0.21	**13.3**	2	0.3	-3.3	24.8	0.00* T
Infradentale	**9.8**	1.34	0.18	**11.9**	1.5	0.22	-2.1	17.6	0.00* T
Supramentale	**11.9**	1.53	0.2	**12.9**	1.85	0.28	-1.1	7.7	0.00* T
Pogonion	**9.8**	2.18	0.29	**11.4**	2.25	0.34	-1.6	14.0	0.00* T
Menton	**7**	1.73	0.24	**9**	2.08	0.33	-2	22.2	0.00* T
Frontal Eminence R	**4.1**	0.89	0.14	**5**	1.23	0.33	-0.9	18.0	0.00* T
Frontal Eminence L	**4.2**	1.11	0.18	**4.9**	1.2	0.32	-0.7	14.2	0.04* T
Mid-supraorbital R	**6.5**	1.27	0.17	**8.6**	1.33	0.2	-2.1	24.4	0.00* T
Mid-supraorbital L	**6.4**	1.37	0.18	**8.7**	1.33	0.2	-2.4	26.4	0.00* T
Mid-infraorbital R	**5.3**	1.47	0.2	**5.8**	1.51	0.23	-0.5	8.6	0.07 M
Mid-infraorbital L	**5.5**	1.58	0.21	**5.9**	1.65	0.25	-0.4	6.7	0.07 M
Malar R	**21.4**	2.57	0.34	**22.8**	2.75	0.41	-1.4	6.1	0.01* T
Malar L	**21.7**	2.38	0.32	**22.9**	2.8	0.42	-1.2	5.2	0.02* T
Lateral Orbital R	**9.1**	1.73	0.23	**8.3**	1.46	0.22	0.8	-9.6	0.02* M
Lateral Orbital L	**9.2**	1.74	0.23	**8.3**	1.36	0.2	0.8	-10.8	0.02* M
Zygion R	**7.8**	1.79	0.24	**9.2**	1.85	0.28	-1.4	15.2	0.00* M
Zygion L	**7.8**	1.9	0.25	**9.1**	1.87	0.28	-1.3	14.2	0.00* M
Supraglenoid R	**10.7**	1.9	0.25	**12.8**	1.57	0.23	-2.1	16.4	0.00* T
Supraglenoid L	**10.7**	1.84	0.25	**12.8**	1.6	0.24	-2.1	16.4	0.00* T
Gonion R	**12.8**	3.65	0.49	**18.3**	5.54	0.83	-5.5	30.0	0.00* M
Gonion L	**13**	3.88	0.52	**17.8**	5.49	0.82	-4.8	26.9	0.00* M
Ectomolare^2 R	**27**	3.62	0.48	**30.3**	3.29	0.49	-3.3	10.8	0.00* M
Ectomolare^2 L	**27.2**	3.64	0.49	**30.2**	3.44	0.51	-2.9	9.9	0.00* M
Occlusal Line R	**20.3**	2.82	0.38	**24.6**	3.17	0.47	-4.4	17.4	0.00* T
Occlusal Line L	**20.4**	2.86	0.38	**24.3**	3.21	0.48	-3.8	16.0	0.00* T
Ectomolare$_2$ R	**24.9**	3.06	0.41	**28.2**	3.96	0.59	-3.4	11.7	0.00* T
Ectomolare$_2$ L	**24.8**	3.2	0.43	**28.4**	3.48	0.52	-3.6	12.6	0.00* T

R – right; L – left; T – *t*-test; M – Mann Whitney

* $P < 0.05$

** ; ***Differences were calculated between female means compared to male means

The FSTT analysis indicates the male sample exhibited higher FSTT values compared to the female sample, except for the orbital lateral, which demonstrated a difference among the averages of 0.8 mm. The major thickness discrepancies between the sexes were found on landmarks the gonion, ectomolare^2, ectomolare$_2$ and occlusal line, in which mean differences were above 3 mm.

Regarding age, an ANOVA was used, on both sexes, to compare averages between the age groups (18 to 30 years, 31 to 40 years and 41 and older). A direct relation between FSTT and ageing process can be seen, particularly among females. The mid-philtrum, prosthion an ectomolare^2 landmarks presented a decrease of FSTT as the age advanced.

Figs 5 and 6 show the comparison by sex among age groups. Quantitatively, a direct relation is noted between age and divergence of FSTT specifically for females, on mid-philtrum, prosthion and ectomolare^2 landmarks, which showed a decrease of FSTT as the age advanced.

Fig 5. FSTT Means: Comparison among Age Groups in Males

Fig 6. FSTT Means: Comparison among Age Groups in Females

Table 4 presents FSTT averages, on each landmark in comparison to the Southeastern sample (SE) [18] and the Midwestern (MW) (this paper), by sex, as well as *P* values (*t* test).

Table 4 – FSTT (in Millimeters) Comparison among SE and MW Regions, in Both Sexes

Landmark	Female					Male				
	SE	MW	Mean Difference (mm)**	Mean Difference (%)***	T Test Sig.	SE	MW	Mean Difference (mm)**	Mean Difference (%)***	T Test Sig.
Supraglabellare	3.4	4.5	-1.1	-24.4	0.00*	4.3	5.5	-1.2	-21.8	0.00*
Glabella	5	4.9	0	0.0	0.94	5.8	5.9	-0.2	-3.4	0.45
Nasion	5.9	6.2	-0.3	-4.8	0.11	7.2	8.1	-1	-12.3	0.00*
Rhinion	1.7	1.7	0	0.0	0.61	1.9	2.3	-0.3	-13.0	0.01*
Mid-Philtrum	12.3	13.1	-0.7	-5.3	0.02*	15.1	15.7	-0.6	-3.8	0.16
Prosthion	9.5	10	-0.5	-5.0	0.17	12.4	13.3	-0.9	-6.8	0.02*
Infradentale	11.3	9.8	1.5	15.3	0.29	11.2	11.9	-0.8	-6.7	0.05*
Supramentale	10.8	11.9	-1.1	-9.2	0.00*	11.4	12.9	-1.5	-11.6	0.00*
Pogonion	9.4	9.8	-0.4	-4.1	0.42	10.8	11.4	-0.7	-6.1	0.21
Menton	6.9	7	-0.1	-1.4	0.87	8.6	9	-0.4	-4.4	0.38
Frontal Eminence R	3.5	4.1	-0.6	-14.6	0.00*	4.4	5	-0.6	-12.0	0.09
Frontal Eminence L	3.4	4.2	-0.8	-19.0	0.00*	4.5	4.9	-0.5	-10.2	0.21
Mid-supraorbital R	6.2	6.5	-0.3	-4.6	0.22	7.3	8.6	-1.3	-15.1	0.00*
Mid-supraorbital L	6.1	6.4	-0.2	-3.1	0.33	7.2	8.7	-1.6	-18.4	0.00*
Mid-infraorbital R	5	5.3	-0.3	-5.7	0.32	5.4	5.8	-0.4	-6.9	0.16
Mid-infraorbital L	4.8	5.5	-0.6	-10.9	0.03	5.4	5.9	-0.5	-8.5	0.07
Malar R	19.4	21.4	-2	-9.3	0.00*	20.3	22.8	-2.5	-11.0	0.00*
Malar L	18.7	21.7	-3	-13.8	0.00*	20.3	22.9	-2.6	-11.4	0.00*
Lateral Orbital R	9	9.1	-0.1	-1.1	0.76	7.5	8.3	-0.8	-9.6	0.01*
Lateral Orbital L	10.4	9.2	1.2	13.0	0.38	7.3	8.3	-1	-12.0	0.00*
Zygion R	7.4	7.8	-0.4	-5.1	0.27	8.1	9.2	-1.1	-12.0	0.01*
Zygion L	7.5	7.8	-0.3	-3.8	0.43	7.8	9.1	-1.4	-15.4	0.00*
Supraglenoid R	10	10.7	-0.7	-6.5	0.06	11.4	12.8	-1.4	-10.9	0.00*
Supraglenoid L	9.9	10.7	-0.8	-7.5	0.04*	11.1	12.8	-1.6	-12.5	0.00*
Gonion R	13.2	12.8	0.3	2.3	0.66	16.9	18.3	-1.4	-7.7	0.21
Gonion L	13.2	13	0.2	1.5	0.73	17.2	17.8	-0.6	-3.4	0.55
Ectomolare^2 R	26	27	-1	-3.7	0.11	28.2	30.3	-2.2	-7.3	0.01*
Ectomolare^2 L	26.3	27.2	-0.9	-3.3	0.17	28.2	30.2	-1.9	-6.3	0.01*
Occlusal Line R	20.1	20.3	-0.2	-1.0	0.73	22.8	24.6	-1.8	-7.3	0.01*
Occlusal Line L	20.4	20.4	0	0.0	0.93	23	24.3	-1.3	-5.3	0.07
Ectomolare$_2$ R	23.5	24.9	-1.3	-5.2	0.03*	25.1	28.2	-3.1	-11.0	0.00*
Ectomolare$_2$ L	24	24.8	-0.8	-3.2	0.18	25.5	28.4	-2.9	-10.2	0.00*

*$P < 0.05$

; * Differences calculated between the averages of SE sample compared to MW sample.

Discussion

FSTT is one of the most studied subjects in the FFR field [22]. It provides quantitative data for the technique [13], but ~~it still has many uncovered issues that justify researchers' continuous interest~~. Studies have confirmed that FSTT measures obtained by using CBCT result in reliable and reproducible data [7,18,23,24].

Several studies have verified the presence of FSTT variances among different populations, suggesting the importance of using specific values for each one [8,10–12,25,26]. Some of these studies compared data of FSTT averages that were obtained with different measurement protocols with inevitable bias to those conclusions [13]. This may explain why there are other studies that contradict this hypothesis and do not support that specific FSTTs are needed for different populations for the FFR [27–29]. The FSTT measurement method is important to the analysis of the specificity among different studies [29]. Our study was able to compare regional FSTT using the same method from a previous study, eliminating the method variant.

The concordance results from the interexaminer ICC analysis showed the Beaini protocol [18] can provide reproducibility and that it is recommended for future research. The intra-examiner ICC analysis demonstrated high concordance on the measurements made by the main examiner, in accordance with other studies that utilized the CBCT with the same objective [18,30]. Nevertheless, it was a result of practical experience gained on previous training, which may be necessary and well demonstrated in future studies.

Compared to the previous research [18], that produced a FSTT chart from a Brazilian Southeastern sample, it was clear that the heterogeneity of the Brazilian population is marked by a diverse population affinity that could not be completely made clear [2]. Analyzing the average of the differences among the Southeastern and Midwestern samples, the negative variation shows a predominance of deeper FSTTs among the Midwestern sample when compared to the Southeastern sample.

Considering the differences related to sex in the samples of the Southeastern [18] and Midwestern regions (this paper), statistical differentiation was observed. In males, differences were more evident in 5 landmarks on the midline and 8 bilateral points. Quantitatively, that variance did not surpass 2.5 mm, except the ectomolare$_2$ with 3.1 mm. Proportionally, it varie between 10 to 15% in most cases with a few landmarks showing

higher values. Within females, studies indicate fewer divergent landmarks (3 on the midline and 8 bilateral) where only malar showed a difference above 2.5 mm (3.0 mm). These results reveal high compatibility among the samples, considering that errors lower than 2.5 mm, may result in high correspondence on a facial pool [31] and differences between 2.5 ≤ 2.9 mm have minimal practical impact [32]

Whether reconstructions should match local facial traits is not under debate, and the use of FSTT tables of a specific ancestry FSTT charts may provide better FFR. However, local changes should not be exclusively related to FSTT [33]. Eyes, lips, nose, and skin features could also be relevant in the differentiation of people from these 2 different areas in Brazil.

Investigating further into our results, they support that previous to the FFR, a careful anthropological evaluation of the skull regarding sex, age and ancestry variance remain necessary [6] because it provides important information to the forensic professional. However, the use of FSTT mean values divided by sex, age, and ancestry is no guarantee ~~to lead to~~ better recognition rates, and awareness of the complexity of soft tissue displacement caused by aging [34]. The assessment of the age variant was originally made to reduce sample distribution bias when compared to the Southeastern sample [18] but it presented interesting results. Our results

show that attention to this matter should not be neglected on reconstructed faces [27].

The same was planned to verify sex differences because the Southeastern research showed statistically significant changes in STT related to sex. In this research, in agreement with previous studies [18,35–37], the analysis among sexes revealed males tend to possess higher depths than females, excepting the lateral orbital landmark, in which it has been observed a mean difference of 0.8 mm.

However, such statistical difference may not be the only reason for sexual dimorphism features [9,36,38]. Landmarks showed FSTT averages variate thickness within a small quantitative interval (<6% average), leaving sex variance among the individuals to skull formation [38].

The variation between males and females was no greater than 3 mm in most points. Only 4 landmarks exhibited greater discrepancies and those were located at anatomical regions most influenced by the BMI [37], such as gonion (5.5 mm), ectomare^2 (3.3 mm), ectomloare$_2$ (3.6 mm) and occlusal line (4.4 mm). Hence, sexual dimorphism was not clearly evidenced in the variation of depths of soft tissue. Despite the statistical differences, the authors found that male and female individuals appear to share similar averages [38]. The human skull is the third best reference for determining

sex, after the pelvic bone and postcranial dimensions [39]. Therefore, the differences verified in this study indicate that sexual dimorphism is probably based, mainly, on the skull morphology.

Multiple facial morphological and physiological alterations act on the ageing process of the face. Its beginning and progression vary between individuals, sexes, and ancestries [40]. In this study, meaningful differences were observed in the FSTT among the age group, notably in females, being the landmarks located on the medium and inferior face. The FSTT averages of the landmarks mid-philtrum, prosthion and ectomolare[2] tend to decrease progressively with age, reproducing the morphological alteration patterns that occur in facial ageing, demonstrating a coherence among the data. With the advancing of age, the average face volume is permanently lost from both from superficial and deep fat tissue [41], and a redistribution of this volume occurs that causes slenderizing of the superior lip more than it does in the inferior one [42]. Bone remodeling in that region results in a decrease of the overlying soft tissue support area. This phenomenon, along with the effect of gravity, characterizes the average ageing of the face [34,43].

Conclusions

The measurements analyzed in this research, when compared to those collected in the Southeast region of Brazil, have statistically differed

~~in~~ many landmarks. However, observing the averages in a specific FSTT chart, indicated that regional differences between the 2 samples are quantitatively small and they should have minimal influence on facial ~~soft tissue thicknesses.~~ Therefore, the use of specific FSTT charts is not necessary when performing FFR of individuals from ~~Brazil's~~ Southeast and Midwest.

References

1. Wilkinson C. Facial reconstruction – anatomical art or artistic anatomy? J Anat. 2010;216: 235–250. doi:10.1111/j.1469-7580.2009.01182.x
2. Wilkinson C. Computerized forensic facial reconstruction : A review of current systems. Forensic Sci Med Pathol. 2005;1: 173–177. doi:10.1385/FSMP:1:3:173
3. Wilkinson C. Facial identification of the dead. J Anat. 2008 [cited 29 Jun 2020]. doi:10.1111/j.1469-7580.2008.00972.x
4. Fernandes CMS, da Costa Serra M, da Silva JVL, Yoshito Noritomi P, de Sena Pereira FDA, Melani RFH. Tests of one Brazilian facial reconstruction method using three soft tissue depth sets and familiar assessors. Forensic Sci Int. 2012;214: 211.e1-211.e7. doi:10.1016/j.forsciint.2011.08.017
5. Stephan CN, Caple JM, Guyomarc'h P, Claes P. An overview of the latest developments in facial imaging. Forensic Sci Res. 2019;4: 10–28. doi:10.1080/20961790.2018.1519892
6. de Almeida NH, Michel-Crosato E, de Paiva LAS, Biazevic MGH. Facial soft tissue thickness in the Brazilian population: new reference data and anatomical landmarks. Forensic Sci Int. 2013;231: 404.e1–7. doi:10.1016/j.forsciint.2013.05.024
7. Fourie Z, Damstra J, Gerrits PO, Ren Y. Accuracy and reliability of facial soft tissue depth measurements using cone beam computer tomography. Forensic Sci Int. 2010;199: 9–14. doi:10.1016/j.forsciint.2010.02.018
8. Cavanagh D, Steyn M. Facial reconstruction: Soft tissue thickness values for South African black females. Forensic Sci Int. 2011;206: 215.e1-215.e7. doi:10.1016/j.forsciint.2011.01.009

9. Tedeschi-Oliveira SV, Melani RFH, de Almeida NH, de Paiva LAS. Facial soft tissue thickness of Brazilian adults. Forensic Sci Int. 2009;193: 127.e1-127.e7. doi:10.1016/j.forsciint.2009.09.002

10. Bulut O, Sipahioglu S, Hekimoglu B. Facial soft tissue thickness database for craniofacial reconstruction in the Turkish adult population. Forensic Sci Int. 2014;242: 44–61. doi:10.1016/j.forsciint.2014.06.012

11. Codinha S. Facial soft tissue thicknesses for the Portuguese adult population. Forensic Sci Int. 2009;184: 80.e1-80.e7. doi:10.1016/j.forsciint.2008.11.011

12. De Donno A, Sablone S, Lauretti C, Mele F, Martini A, Introna F, et al. Facial approximation: Soft tissue thickness values for Caucasian males using cone beam computer tomography. Leg Med. 2019;37: 49–53. doi:10.1016/j.legalmed.2019.01.002

13. Stephan CN, Simpson EK. Facial Soft Tissue Depths in Craniofacial Identification (Part I): An Analytical Review of the Published Adult Data*. J Forensic Sci. 2008 [cited 26 Jun 2020]. doi:10.1111/j.1556-4029.2008.00852.x

14. Parra FC, Amado RC, Lambertucci JR, Rocha J, Antunes CM, Pena SDJ. Color and genomic ancestry in Brazilians. Proc Natl Acad Sci. 2003;100: 177–182. doi:10.1073/pnas.0126614100

15. Gonzales PS, Machado CEP, Michel-Crosato E. Photoanthropometry of the Face in the Young White Brazilian Population. Braz Dent J. 2018;29: 619–623. doi:10.1590/0103-6440201802027

16. Jurda M, Urbanová P. Sex and ancestry assessment of Brazilian crania using semi-automatic mesh processing tools. Leg Med. 2016;23: 34–43. doi:10.1016/j.legalmed.2016.09.004

17. Cunha E, Lopez-Capp TT, Inojosa R, Marques SR, Moraes LOC, Liberti E, et al. The Brazilian identified human osteological collections. Forensic Sci Int. 2018;289: 449.e1-449.e6. doi:10.1016/j.forsciint.2018.05.040

18. Beaini TL, Miamoto P, Duailibi-Neto EF, Tedeschi-Oliveira SV, Chilvarquer I, Melani RFH. Facial soft tissue depth measurements in cone-beam computed tomography: A study of a Brazilian sample. Leg Med. 2021;50: 101866. doi:10.1016/j.legalmed.2021.101866

19. Dias PEM, Beaini TL, Melani RFH. Evaluation of osifix software with craniofacial anthropometric purposes. J Res Dent. 2013;1: 351. doi:10.19177/jrd.v1e42013351-367

20. Rhine JS, Campbell HR. Thickness of facial tissues in American blacks. J Forensic Sci. 1980;25: 847–858.

21. Haukoos JS. Advanced Statistics: Bootstrapping Confidence Intervals for Statistics with “Difficult” Distributions. Acad Emerg Med. 2005;12: 360–365. doi:10.1197/j.aem.2004.11.018

22. Stephan CN, Munn L, Caple J. Facial soft tissue thicknesses: Noise, signal, and P. Forensic Sci Int. 2015;257: 114–122. doi:10.1016/j.forsciint.2015.07.051

23. Hwang H-S, Kim K, Moon D-N, Kim J-H, Wilkinson C. Reproducibility of Facial Soft Tissue Thicknesses for Craniofacial Reconstruction Using Cone-Beam CT Images: FACIAL SOFT TISSUE THICKNESS USING CBCT IMAGES. J Forensic Sci. 2012;57: 443–448. doi:10.1111/j.1556-4029.2011.02007.x

24. Hwang H-S, Choe S-Y, Hwang J-S, Moon D-N, Hou Y, Lee W-J, et al. Reproducibility of Facial Soft Tissue Thickness Measurements Using Cone-Beam CT Images According to the Measurement Methods. J Forensic Sci. 2015;60: 957–965. doi:10.1111/1556-4029.12766

25. Phillips VM, Smuts NA. Facial reconstruction: utilization of computerized tomography to measure facial tissue thickness in a mixed racial population. Forensic Sci Int. 1996;83: 51–59. doi:10.1016/0379-0738(96)02010-5

26. Baillie LJ, Mirijali SA, Niven BE, Blyth P, Dias GJ. Ancestry and BMI Influences on Facial Soft Tissue Depths for A Cohort of Chinese and Caucasoid Women in Dunedin, New Zealand. J Forensic Sci. 2015;60: 1146–1154. doi:10.1111/1556-4029.12799

27. Lee W-J, Wilkinson CM, Hwang H-S, Lee S-M. Correlation Between Average Tissue Depth Data and Quantitative Accuracy of Forensic Craniofacial Reconstructions Measured by Geometric Surface Comparison Method. J Forensic Sci. 2015;60: 572–580. doi:10.1111/1556-4029.12726

28. Thiemann N, Keil V, Roy U. In vivo facial soft tissue depths of a modern adult population from Germany. Int J Legal Med. 2017;131: 1455–1488. doi:10.1007/s00414-017-1581-y

29. Guyomarc'h P, Santos F, Dutailly B, Coqueugniot H. Facial soft tissue depths in French adults: variability, specificity and estimation. Forensic Sci Int. 2013;231: 411.e1–10. doi:10.1016/j.forsciint.2013.04.007

30. Baccarin LS. Análise da relação entre as espessuras médias de tecidos moles da face de crianças e adultos para reconstrução facial forense. Doutorado, Universidade de São Paulo. 2019.

31. Stephan CN. Facial Approximation-From Facial Reconstruction Synonym to Face Prediction Paradigm. J Forensic Sci. 2015;60: 566–571. doi:10.1111/1556-4029.12732

32. Briers N, Steyn M. Re-assessment of South African juvenile facial soft tissue thickness data for craniofacial approximation: A comparative analysis using central tendency statistics. Forensic Sci Int. 2018;291: 280.e1-280.e13. doi:10.1016/j.forsciint.2018.08.008

33. Wilkinson C, Neave R, Smith D. How important to facial reconstruction are the correct ethnic group tissue depths? Proceedings of the 10th Conference of the International Association of Craniofacial Identification. Bari, Italy: University of Bari; 2002. pp. 111–21.

34. Buchanan DR, Wulc AE. Contemporary Thoughts on Lower Eyelid/Midface Aging. Clin Plast Surg. 2015;42: 1–15. doi:10.1016/j.cps.2014.09.003

35. Farias Gomes A, Moreira DD, Zanon MF, Groppo FC, Haiter-Neto F, Freitas DQ. Soft tissue thickness in Brazilian adults of different skeletal classes and facial types: A cone beam CT – Study. Leg Med. 2020;47: 101743. doi:10.1016/j.legalmed.2020.101743

36. Dong Y, Huang L, Feng Z, Bai S, Wu G, Zhao Y. Influence of sex and body mass index on facial soft tissue thickness measurements of the northern Chinese adult population. Forensic Sci Int. 2012;222: 396.e1-396.e7. doi:10.1016/j.forsciint.2012.06.004

37. De Greef S, Vandermeulen D, Claes P, Suetens P, Willems G. The influence of sex, age and body mass index on facial soft tissue depths. Forensic Sci Med Pathol. 2009;5: 60–65. doi:10.1007/s12024-009-9085-9

38. Stephan CN, Norris RM, Henneberg M. Does sexual dimorphism in facial soft tissue depths justify sex distinction in craniofacial identification? J Forensic Sci. 2005;50: 513–518.

39. Nikita E, Michopoulou E. A quantitative approach for sex estimation based on cranial morphology. Am J Phys Anthropol. 2018;165: 507–517. doi:10.1002/ajpa.23376

40. Cotofana S, Gotkin RH, Ascher B, Morozov SP, Gombolevsky VA, Laipan AS, et al. Calvarial Volume Loss and Facial Aging: A Computed Tomographic (CT)-Based Study. Aesthet Surg J. 2018;38: 1043–1051. doi:10.1093/asj/sjy096

41. Boehm LM, Morgan A, Hettinger P, Matloub HS. Facial Aging: A Quantitative Analysis of Midface Volume Changes over 11 Years. Plast Reconstr Surg. 2021;147: 319–327. doi:10.1097/PRS.0000000000007518

42. Ko AC, Korn BS, Kikkawa DO. The aging face. Surv Ophthalmol. 2017;62: 190–202. doi:10.1016/j.survophthal.2016.09.002

43. Mendelson B, Wong C-H. Changes in the Facial Skeleton With Aging: Implications and Clinical Applications in Facial Rejuvenation. Aesthetic Plast Surg. 2012;36: 753–760. doi:10.1007/s00266-012-9904-3

Figure 1

Click here to access/download;Figure;Fig1.tif

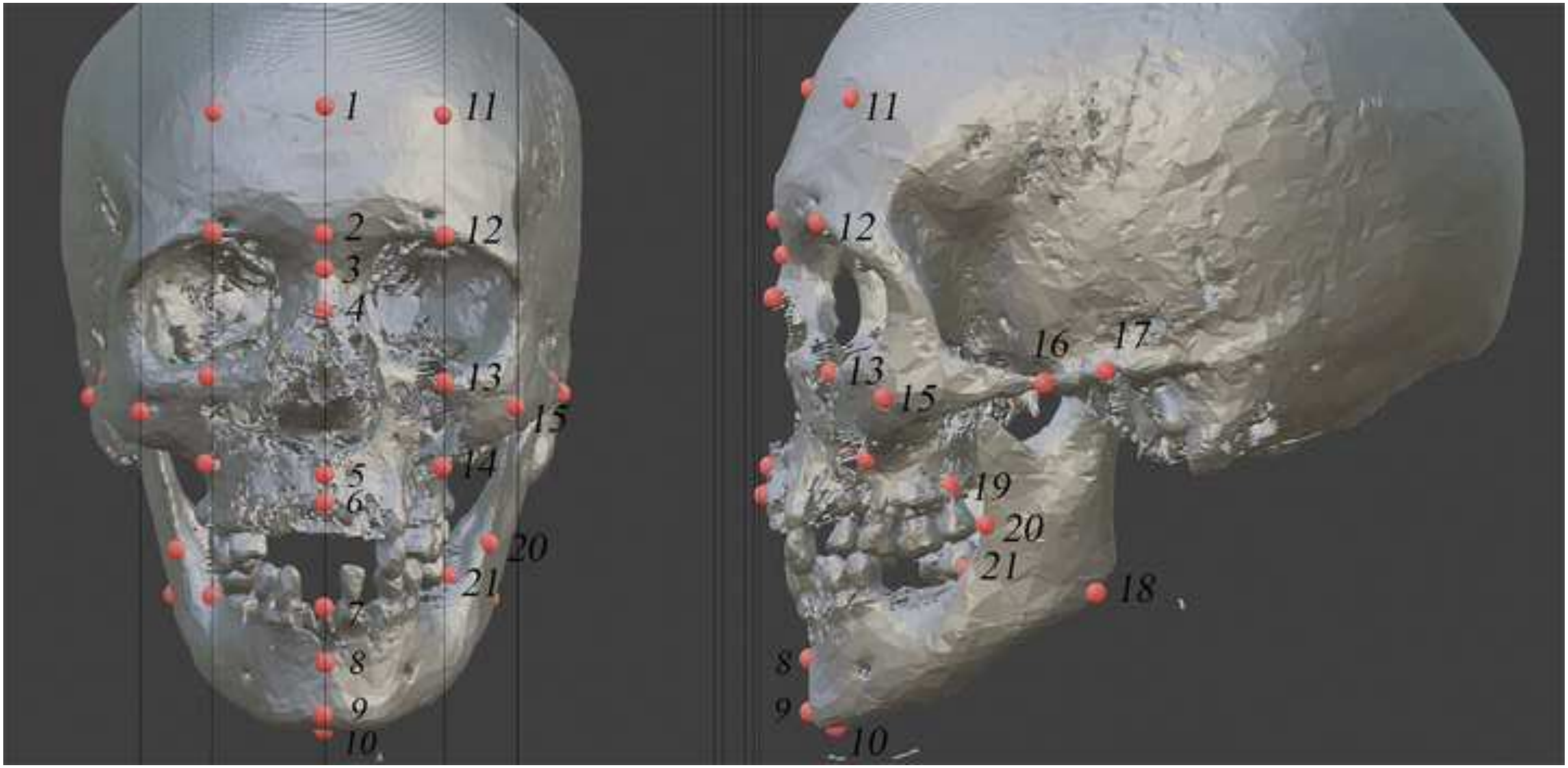

Figure 2

Click here to access/download;Figure;Fig2.tif

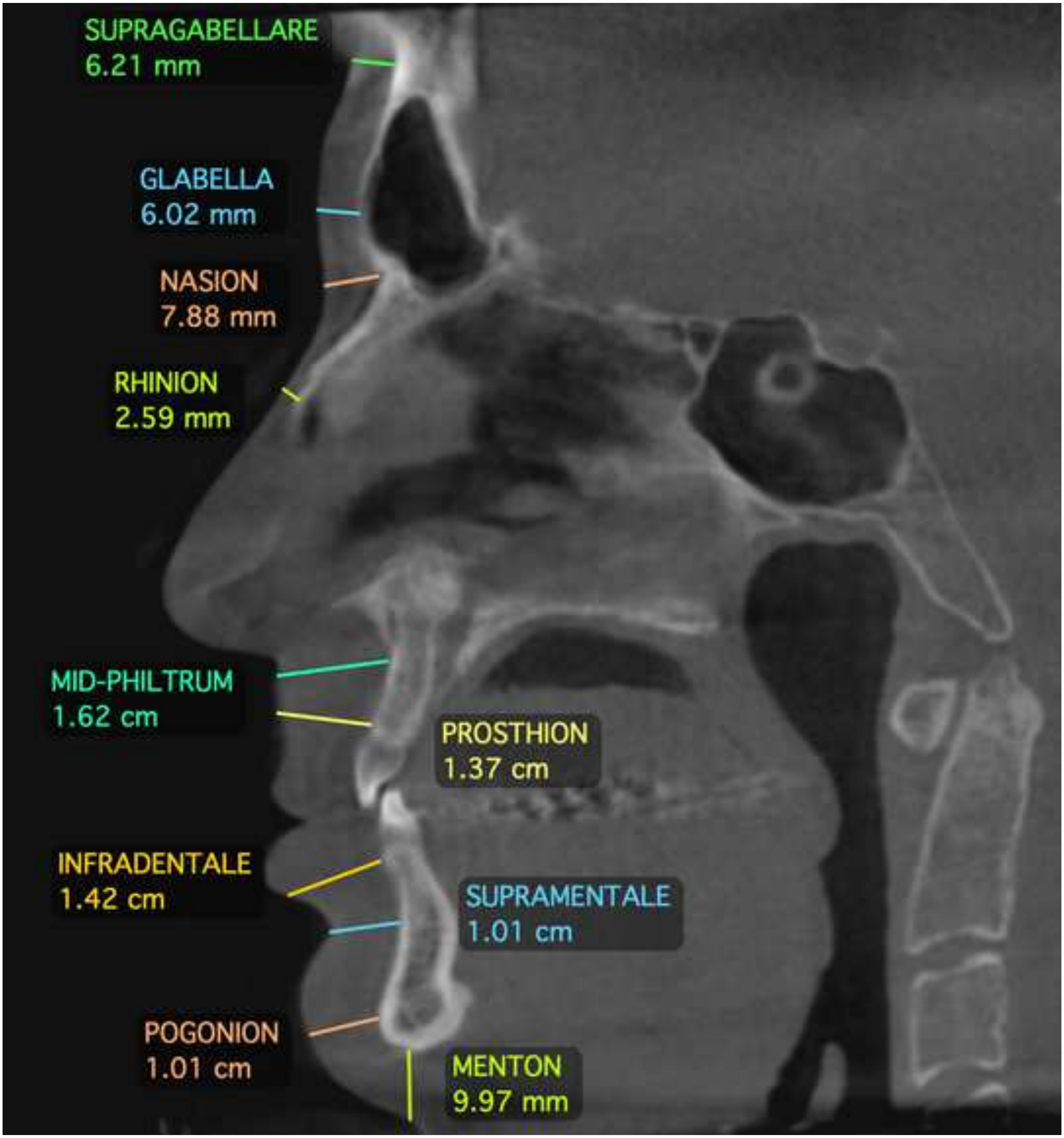

Figure 3

Click here to access/download;Figure;Fig3.tif

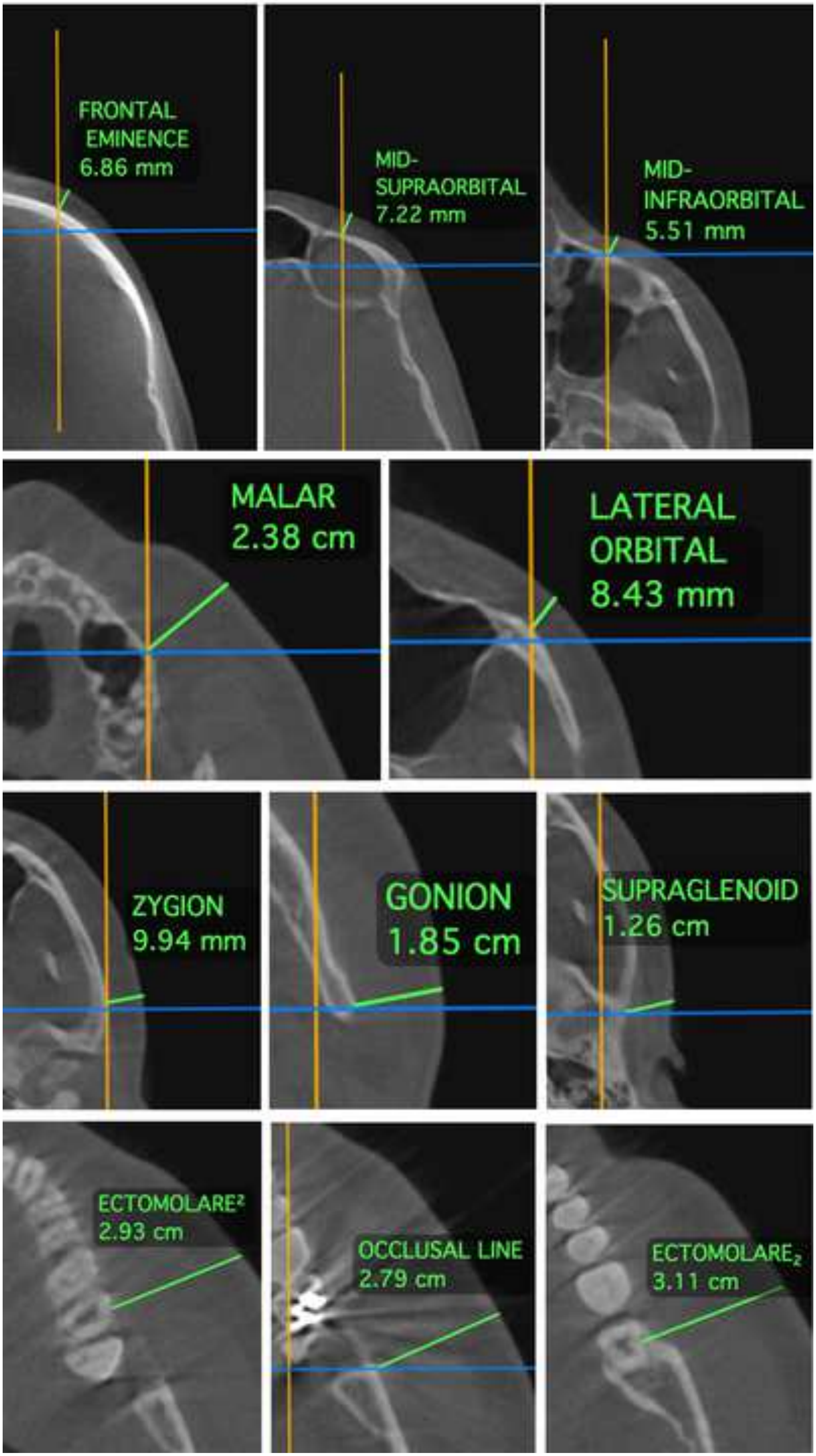

Figure 4

Click here to access/download;Figure;Fig4.tif

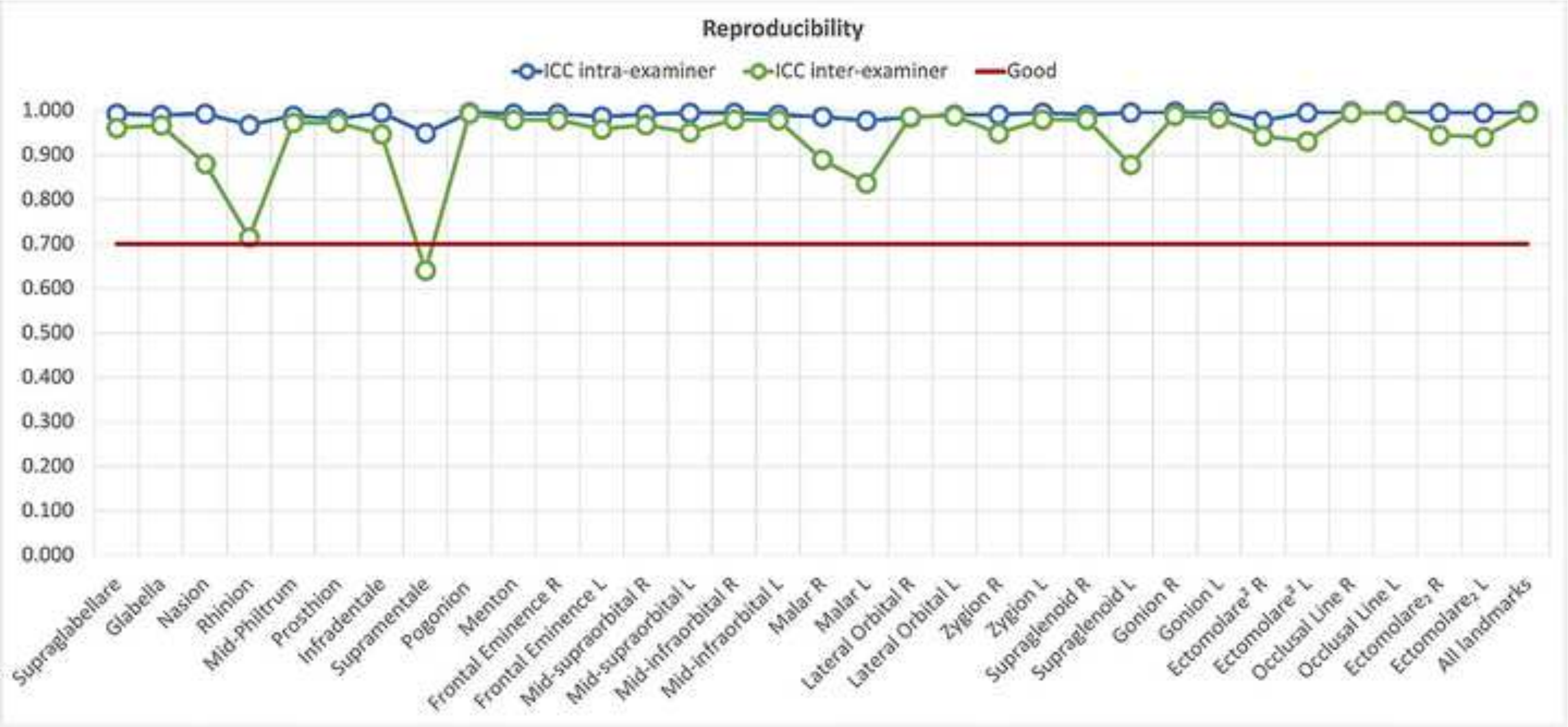

Figure 5

Click here to access/download;Figure;Fig5.tif

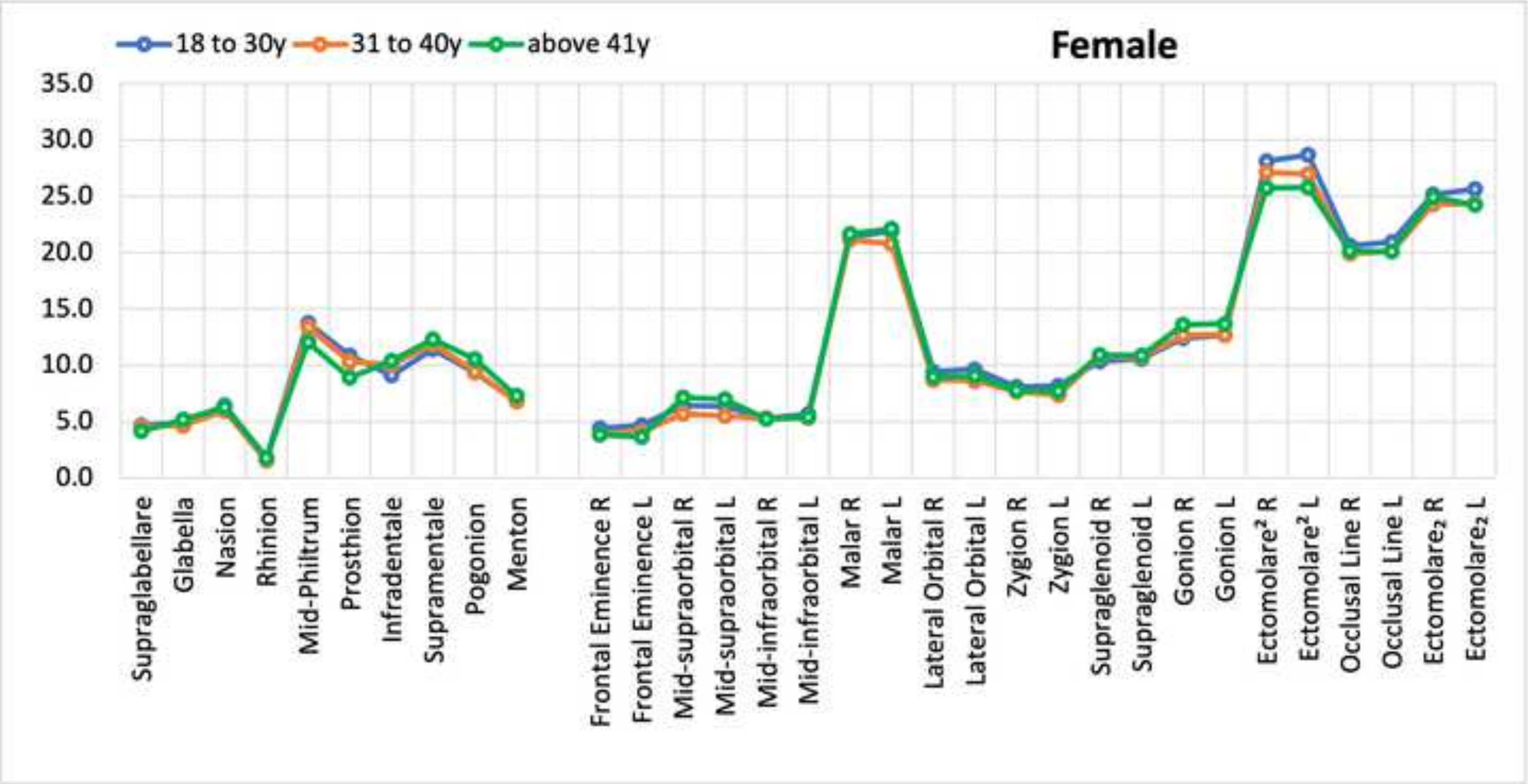

Figure 6

Click here to access/download;Figure;Fig6.tif

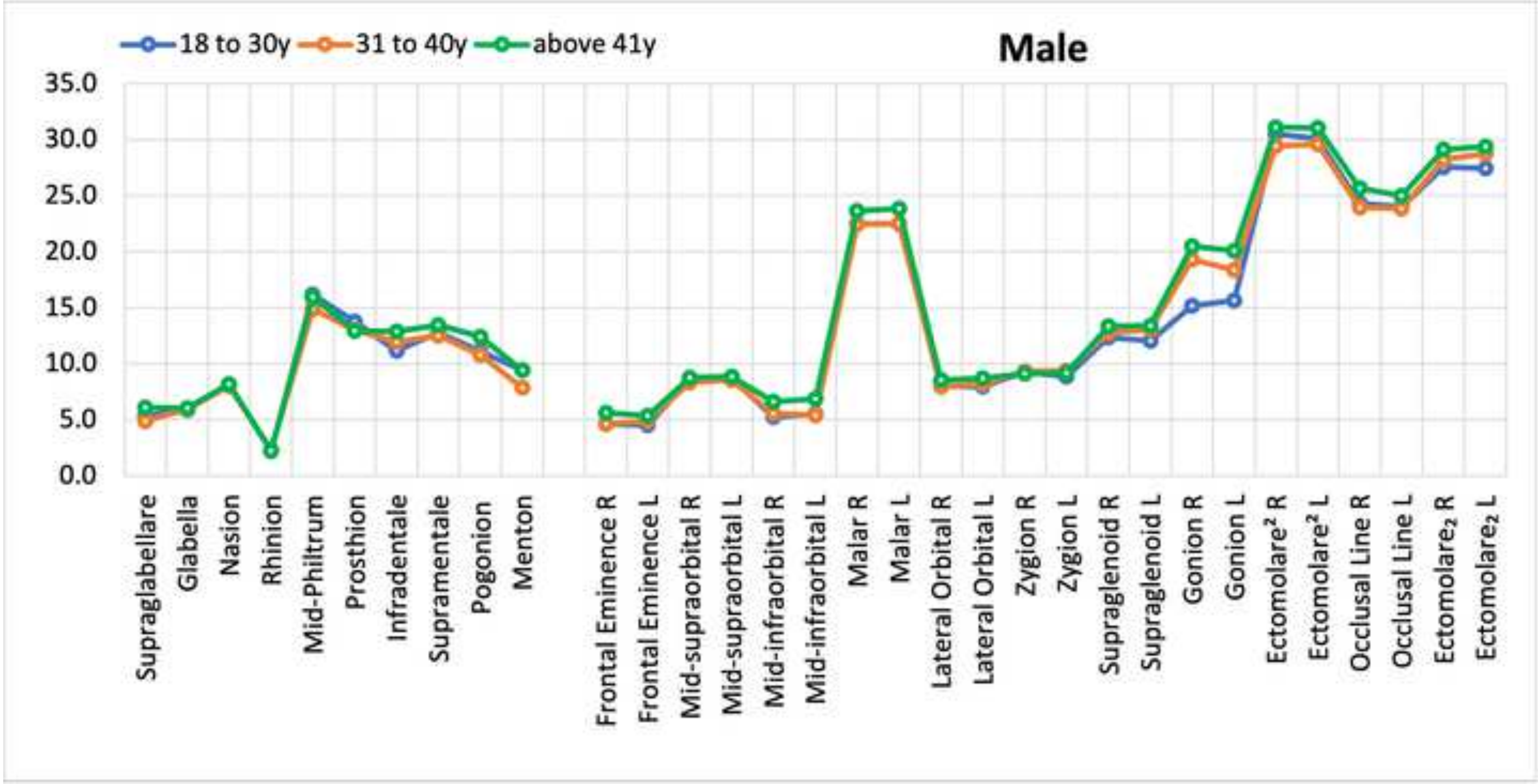

Facial soft tissue thickness in forensic facial reconstruction: impact of regional differences in Brazil

Deisy Satie Moritsugui[1¶*], Flavia Vanessa Greb Fugiwara[1&], Flávia Nicolle Stefani Vassallo[1&], Luiz Eugênio Nigro Mazzilli[1¶], Thiago Leite Beaini[2¶], Rodolfo Francisco Haltenhoff Melani[1¶]

[1] Laboratory of Forensic Anthropology and Odontology (OFLAB), Department of Social Dentistry, School of Dentistry, University of São Paulo, São Paulo, São Paulo, Brazil

[2]Department of Preventive and Social Dentistry, School of Dentistry, Federal University of Uberlândia, Uberlândia, Minas Gerais, Brazil

*Corresponding author

E-mail: dsmoritsugui@usp.br (DSM)

[¶] These authors contributed equally to this work.

[&] These authors also contributed equally to this work.

Abstract

Forensic facial reconstruction aims to assemble and provide the appearance of a face over a skull with the intent of allowing the recognition of that individual, expecting the antemortem information to be presented, and, therefore, making possible the application of primary identification methods. The scientific literature applies reference tables regarding the facial soft tissue thickness (FSTT) in a range of different geographic regions. However, the consensus on its importance or on how to use specific population data related to FSTT is not unanimous. Brazil is formed by geographic regions with diverse environmental characteristics and populations, which are reflected in facial features. This paper aimed to measure and compare FSTT of distinct Brazilian samples to ascertain the need for specific data sets for different regions. A specific protocol for cone beam computed tomography was used to standardize measurement, and it was applied in a sample of 101 exams. The FSTT measurements of a Brazilian population from the Midwest Region was compared to a previous sample from Southeast, which was collected using the same protocol. High compatibility was observed when comparing the averages of FSTT among samples of these two different geographic regions. Regarding age groups, notable differences on the medium and inferior face were observed in females. Minor variances found are unlikely to affect the practice of forensic

facial reconstruction. Facial features, such as eyes, lips, nose, and skin may also be relevant in the differentiation of people from these 2 areas in Brazil. Therefore, concerning the Southeast and Midwest Brazilian regions, the need to apply different data sets is unnecessary.

Keywords: forensic facial reconstruction, forensic facial approximation, human identification, cone beam computed tomography, forensic anthropology.

Introduction

The forensic facial reconstruction (FFR) has an important role in recognizing individuals who are unable to be identified by other methods. Advanced postmortem decomposition process or the lack of antemortem information are its main indication for forensic purposes [1,2]. The technique is based on the assembling of an approximated soft tissue topography over the unknown skull, with the intent of reconstructing its characteristics to be as similar as possible to the individual at the time of his or her death. Once the reconstruction is finished, a public campaign is issued, aiming to illicit a response from the public as to whom the remains might belong. Following this, a formal identification can then take place [1,3,4].

An approximated face is achievable by using various techniques. The 2D representation is based on a picture of the skull, whereas the 3D

representation can be divided into manual or digital methods. The manual method involves direct modelling and sculpture, applying clay or Plasticine over the skull or a model of the skull [1]. More recently, a computerized face sculpture can be generated by using specialized modeling software, and different user interfaces can adapt the procedures to resemble manual modeling. Tablets and the assistance of haptic devices may be used in the 3D context to add soft tissues to a model of the skull [5]. However, independently of the method used, it is imperative to establish parameters and guidance such as the facial soft tissue thickness (FSTT), that serves as reference over specific craniometric landmarks [6], Those FSTT measurements are usually populational-based averages, but from this general information it is often expected to deliver a precise and reliable assistance during FFR [7].

FSTT charts in the scientific literature cover many craniometric landmarks, allowing it to be organized by sex, age, ancestry, BMI, among other facial features considered to be significant in an FFR. Many aspects of the method that seem to be related to the success rate or to the probability of recognition of the reconstructed faces. Studies have indicated that FSTT data are particularly influenced by the population groups under study [8–12], but the debate on the subject has far from achieved a consensus. Therefore, the expert should consider using of FSTT based on the population to which the

body can be correlated by previous anthropological exams because specific FSTT charts could produce better results. However, in those studies, it is remarkable that many describe different methodologies, including the original measurement protocols, the source and type of imaging exam and the relation of regional charts, and the success of the FFR has not yet been established. Hence, some state evidence to justify the need for applying different population data to FFR is insufficient [13], but the doubt still raises questions in situations such as the reality of countries such as Brazil.

The Brazilian territory has continental proportions, and it is divided into geographical regions (north, northeast, southeast, south, and Midwest), each one with its diverse environments and populations.

To understand the local context better, it is important to remember that the original population was based on several Indigenous Brazilian with different anthropological features among the groups found in the territory. The European colonization favored the concept of a mixed ancestry population, mostly influenced by the presence of Portuguese, Dutch and French. After the world wars of the 20th century, the presence of immigrants from Japan, Eastern Europe, the Middle East, Italy and Germany became important [14]. Their distribution was not even among the regions and, therefore, it is clear that each segment of Brazil has its own physical features that represent the dominant population groups, with different characteristics

that can be traced to one's origin [15]. The increasing and continuous lack of ancestral uniformity, as it is observed in Brazil, raises the question considering if the usage of a single STT chart is capable to approximate one's morphological facial features, in this great individual variety context [16].

This study considers that the skull-facial morphology is determined by hereditary features and influenced by the environment [11], affecting the skull and the face. The combination of these anatomical differences makes the individual unique, leading to a wide variation of facial profiles across the country [17]. It justifies deeper investigation and the collection of data from different regions. With the intent of verifying the need for various FSTT charts on reconstruction from each geographic region, this research aimed to measure the FSTT in a Brazilian sample from the Midwest region and to compare to a previous sample from the Southeast region [18] using the same cone-beam computed tomography (CBCT) measuring protocol.

Materials and methods

The sample was composed of 101 CBCT exams of Brazilian subjects from the Midwest region, available in a previously established anonymous data bank in Digital Imaging Communication in Medicine (DICOM) format. Table 1 shows sample distribution.

Table 1 – Sample distribution by sex and age group

Sex<br>Age group	Male	Female	Total
18 to 30 years	18	22	40
31 to 40 years	13	14	27
41 and older	14	20	34
**Total**	45	56	101

The exams were acquired from records of clinical diagnosis, and, therefore, no participant was exposed to ionizing radiation for this research. The image protocol had a 23 cm x 17 cm FOV field of view, allowing the visualization of all included anatomical structures and landmarks from the supraglabellare to menton in the vertical axis and posterior to the supraglenoid structure. The images were obtained with an i-CAT Next Generation tomograph with 17.8 second exposition and voxel of 0.3 mm, and they were exported on Digital Imaging and Communication in Medicine (DICOM) format. CBCT scans allow image acquisitions with the individual in a sitting or standing position. Despite containing stabilizing devices for the head, such as the cephalostat and the chin support, these were not used in our sample, which avoided the effects of compression of facial soft tissues.

The research was approved by the Committee of Ethics in Research of the School of Dentistry of University of São Paulo (CAAE 33433320.8.0000.007 and 33433320.8.0000.0075). The inclusion criteria were established by observing the facial integrity of the research subjects.

Therefore, it included the exams of adult patients (18 and older) with required presence of certain structures: the central incisors and second molars (including both natural and prosthetically rehabilitated elements), no visible facial syndromes (hard or soft tissue), with no severe asymmetry and that had not been submitted to orthognathic surgery. Furthermore, the research included exams of both male and female patients in balanced groups. Thus, the exclusion criteria rejected exams that did not fit all the inclusion criteria or had quality issues that could prejudice the measuring of soft tissues.

The selection and measurements were performed with the assistance of the Horos software (version 3.3.6, 64 bit, Horos Project, Purview, Annapolis, USA) installed on an iMac computer (macOS Catalina – version 10.25.2, a 3,4 GHz Intel Core i7 Quad-Core processor, 16 GB RAM) and the 3D reconstruction tool 3D volume rendering through the skin and bone filters. Horos software is an open access version of OsiriX (DICOM viewer software) and has demonstrated to be a good alternative for craniofacial anthropological analyses, both in hard and soft tissues [19].

The exams were divided by individuals' sex into 3 age groups (from 18 to 30 years old, from 31 to 40 years old, 41 and older) following the original research group divisions [18], whose control sample, from

Southeast Brazilian region, was composed of 100 exams equally distributed among the groups.

The protocol established by Beaini et al. [18] was used to create a standardized measurements of FSTT in CBCT. In this guide, the skull images were positioned with the Frankfurt plan parallel to the horizontal axis to avoid the influence of misplacement on multiplanar view. The measurements of FSTT were executed in the same craniometric landmarks studied by Rhine and Campbell [20]. Thrirty-two craniometric landmarks were measured, 10 located on the midline and 11 bilateral landmarks. Figs 1-3 present the craniometric landmarks and measurement direction, whereas Table 2 describes each landmark and the measurement directions according to the applied protocol.

Fig 1. Craniometric Landmarks Measured

Fig 2. Sagittal Landmarks Measurements

Fig 3. Bilateral Landmarks Measurements

Table 2 – Craniometric Landmarks, Descriptions, and Measurement Direction

	**Landmark**	**Description**	**Measurement direction**
	**Median points**		

1	**Supraglabellare**	Deepest part of the supraglabella fossa in the median plane	Perpendicular to the bone surface
2	**Glabella**	Most projecting median point on lower edge of the frontal bone	Perpendicular to the bone surface
3	**Nasion**	Intersection of the nasofrontal sutures in the median plane	Nasal soft tissue fold
4	**Rhinion**	Most rostral point on the internasal suture	Perpendicular to the bone surface
5	**Mid-Philtrum**	Deepest point of the maxillary alveolar bone	Mid-Philtrum or subnasale
6	**Prosthion**	Median point between the central incisors on the anterior margin of the maxillary alveolar rim	Upper vermilion border
7	**Infradentale**	Median point at the superior tip of the septum between the mandibular central incisors	Lower vermilion border
8	**Supramentale**	Deepest median point in the groove superior to the mental eminence	Chin fold
9	**Pogonion**	Most anterior median point on the mental eminence of the mandible	Perpendicular to the bone surface
10	**Menton**	Most inferior median point of the mental symphysis	Vertical direction or to neck fold
	**Bilateral Points**		
11	**Frontal Eminence**	Most projecting point of the frontal bone, at a line that vertically bisects the orbit	Perpendicular to the bone surface
12	**Mid-supraorbital**	Point on the anterior aspect of the superior orbital rim, at a line that vertically bisects the orbit	Orthoradial direction
13	**Mid-infraorbital**	Point on the anterior aspect of the inferior orbital rim, at a line that vertically bisects the orbit	Orthoradial direction
14	**Malar**	Intersection of the maxillary alveolar process and zygomatic process, at a line that vertically bisects the orbit	Perpendicular to the bone surface
15	**Lateral Orbital**	On the center of the zygomatic bone, at a line that vertically crosses the lateral orbital margin	Orthoradial direction
16	**Zygion**	The most lateral point on the zygomatic arch	Orthoradial direction
17	**Supraglenoid**	Most rostral point on the superior rim of the glenoid cavity	Orthoradial direction
18	**Gonion**	Point on the rounded margin of the angle of the mandible, bisecting two lines one following vertical margin of ramus and one following horizontal margin of the corpus of mandible	Perpendicular to the bone surface

**19**	**Ectomolare^2**	Most lateral point on the buccal alveolar margin, at the center of the superior second molar position	Orthoradial direction
**20**	**Occlusal Line**	Most anterior point on the mandibular ramus, where the occlusal line meets de mandible	Orthoradial direction
**21**	**Ectomolare$_2$**	Most lateral point on the buccal alveolar margin, at the center of the inferior second molar position	Orthoradial direction

The DICOM files from CBCT were imported into the Horos software, according to the protocol by Beaini et al. [18], a window level of 500 e window width of 3500 were applied to all exams to favor the visualization of both hard and soft tissue limits. The acquired volume was reconstructed on a 3D MPR window (multiplanar reconstruction) and visualized with the aid of a MIP tool (maximum intensity projection) to perform the volume repositioning to the Frankfurt plane reference, as previously mentioned.

An initial calibration was made in the form of inter- and intraobserver tests. The measurements for this research were collected only with a high concordance between observers. The evaluation and adjustment of inter and intra-examiners were achieved by testing the 32 landmarks in 15 exams (15% of the sample) and testing the intraclass correlation coefficient (ICC) for each point. Regarding intra-examiner assessment, the main examiner measured the 15 exams twice, considering a 1 week time interval

between the measurements. The main examiner's previous training of the method aimed to agree with the original examiner of the compared research.

The data were organized in a spreadsheet, using the software Microsoft Excel, and statistical analysis was performed with aid of the SPSS statistic pack, establishing a reliability level of 95%.

Using the Shapiro-Wilk test, the normality distribution sample was checked for every landmark, with $P < 0.05$, which implies alteration of normality. In the nonnormal distribution landmarks, the bootstrap method was used (1000 resampling – 95% CI BCa) to obtain reliable average values.

To search for sex related differences, the *t*-test was applied on landmarks with normal distribution, whereas the Mann-Whitney test was used with nonnormal distribution data. The variance analysis from a one-way ANOVA was applied to investigate any differences among the average FSTT, by landmark, on the three age groups (18 to 30 years, 31 to 40 years and 41 and older).

Concerning the regional differences, the research data were compared to the raw data of the Southeast region [18] to which full access was granted. A *t*-test was applied on the comparison of FSTT averages among the Southeastern [18] and Midwestern (this study) regions, for both sexes.

Results

The concordance of the measurements verified by the ICC according to each landmark measurement revealed intra- and interexaminer agreement above 95%, demonstrating reliability among the measurements. Fig 4 presents the individual ICC records.

Fig 4. Concordance Level for Each Landmark in Intra- and Interobserver Analysis

For male subjects, the Shapiro-Wilk test revealed that, from the 32 analyzed landmarks, 2 presented nonnormal distribution (lateral orbital and ectomolare^2). Female subjects presented nonnormal distribution at the landmarks rhinion, mid-infraorbital, zygion and gonion. In nonnormal distribution landmarks, the bootstrap method (1000 resamples – 95% CI BCa) was used to obtain reliable mean values. Such statistical procedure assigns consistent results, through the process of random resampling, because the process tends to correct normality deviations of the sample [21].

Table 3 exhibits the FSTT averages as the main result of this research. It has been divided by sex with SD, SE and *P* values, corresponding to each test, verifying statistically significant differences between the female and male averages, after bootstrapping procedure.

Table 3 – Mean FSTT (Millimeters and Percentage), SD, and SE Divided by Sex

	Female			Male			Mean difference (mm) **	Mean difference (%) ***	*P* value
	Mean	SD	SE	Mean	SD	SE			
Supraglabella	**4.5**	0.97	0.14	**5.5**	1.14	0.23	-1	18.1	0.00* T
Glabella	**4.9**	0.77	0.1	**5.9**	0.95	0.14	-1	16.9	0.00* T
Nasion	**6.2**	0.97	0.13	**8.1**	1.24	0.18	-1.9	23.4	0.00* T
Rhinion	**1.7**	0.43	0.06	**2.3**	0.58	0.09	-0.5	26.0	0.00* M
Mid-Philtrum	**13.1**	1.82	0.24	**15.7**	2.22	0.33	-2.7	16.5	0.00* T
Prosthion	**10**	1.58	0.21	**13.3**	2	0.3	-3.3	24.8	0.00* T
Infradentale	**9.8**	1.34	0.18	**11.9**	1.5	0.22	-2.1	17.6	0.00* T
Supramentale	**11.9**	1.53	0.2	**12.9**	1.85	0.28	-1.1	7.7	0.00* T
Pogonion	**9.8**	2.18	0.29	**11.4**	2.25	0.34	-1.6	14.0	0.00* T
Menton	**7**	1.73	0.24	**9**	2.08	0.33	-2	22.2	0.00* T
Frontal Eminence R	**4.1**	0.89	0.14	**5**	1.23	0.33	-0.9	18.0	0.00* T
Frontal Eminence L	**4.2**	1.11	0.18	**4.9**	1.2	0.32	-0.7	14.2	0.04* T
Mid-supraorbital R	**6.5**	1.27	0.17	**8.6**	1.33	0.2	-2.1	24.4	0.00* T
Mid-supraorbital L	**6.4**	1.37	0.18	**8.7**	1.33	0.2	-2.4	26.4	0.00* T
Mid-infraorbital R	**5.3**	1.47	0.2	**5.8**	1.51	0.23	-0.5	8.6	0.07 M
Mid-infraorbital L	**5.5**	1.58	0.21	**5.9**	1.65	0.25	-0.4	6.7	0.07 M
Malar R	**21.4**	2.57	0.34	**22.8**	2.75	0.41	-1.4	6.1	0.01* T
Malar L	**21.7**	2.38	0.32	**22.9**	2.8	0.42	-1.2	5.2	0.02* T
Lateral Orbital R	**9.1**	1.73	0.23	**8.3**	1.46	0.22	0.8	-9.6	0.02* M
Lateral Orbital L	**9.2**	1.74	0.23	**8.3**	1.36	0.2	0.8	-10.8	0.02* M
Zygion R	**7.8**	1.79	0.24	**9.2**	1.85	0.28	-1.4	15.2	0.00* M
Zygion L	**7.8**	1.9	0.25	**9.1**	1.87	0.28	-1.3	14.2	0.00* M
Supraglenoid R	**10.7**	1.9	0.25	**12.8**	1.57	0.23	-2.1	16.4	0.00* T
Supraglenoid L	**10.7**	1.84	0.25	**12.8**	1.6	0.24	-2.1	16.4	0.00* T
Gonion R	**12.8**	3.65	0.49	**18.3**	5.54	0.83	-5.5	30.0	0.00* M
Gonion L	**13**	3.88	0.52	**17.8**	5.49	0.82	-4.8	26.9	0.00* M
Ectomolare^2 R	**27**	3.62	0.48	**30.3**	3.29	0.49	-3.3	10.8	0.00* M
Ectomolare^2 L	**27.2**	3.64	0.49	**30.2**	3.44	0.51	-2.9	9.9	0.00* M
Occlusal Line R	**20.3**	2.82	0.38	**24.6**	3.17	0.47	-4.4	17.4	0.00* T
Occlusal Line L	**20.4**	2.86	0.38	**24.3**	3.21	0.48	-3.8	16.0	0.00* T
Ectomolare$_2$ R	**24.9**	3.06	0.41	**28.2**	3.96	0.59	-3.4	11.7	0.00* T
Ectomolare$_2$ L	**24.8**	3.2	0.43	**28.4**	3.48	0.52	-3.6	12.6	0.00* T

R – right; L – left; T – *t*-test; M – Mann Whitney

* $P < 0.05$

** ; ***Differences were calculated between female means compared to male means

The FSTT analysis indicates the male sample exhibited higher FSTT values compared to the female sample, except for the orbital lateral, which demonstrated a difference among the averages of 0.8 mm. The major thickness discrepancies between the sexes were found on landmarks the gonion, ectomolare^2, ectomolare$_2$ and occlusal line, in which mean differences were above 3 mm.

Regarding age, an ANOVA was used, on both sexes, to compare averages between the age groups (18 to 30 years, 31 to 40 years and 41 and older). A direct relation between FSTT and ageing process can be seen, particularly among females. The mid-philtrum, prosthion an ectomolare^2 landmarks presented a decrease of FSTT as the age advanced.

Figs 5 and 6 show the comparison by sex among age groups. Quantitatively, a direct relation is noted between age and divergence of FSTT specifically for females, on mid-philtrum, prosthion and ectomolare^2 landmarks, which showed a decrease of FSTT as the age advanced.

Fig 5. FSTT Means: Comparison among Age Groups in Males

Fig 6. FSTT Means: Comparison among Age Groups in Females

Table 4 presents FSTT averages, on each landmark in comparison to the Southeastern sample (SE) [18] and the Midwestern (MW) (this paper), by sex, as well as *P* values (*t* test).

Table 4 – FSTT (in Millimeters) Comparison among SE and MW Regions, in Both Sexes

Landmark	Female					Male				
	SE	MW	Mean Difference (mm)**	Mean Difference (%)***	T Test Sig.	SE	MW	Mean Difference (mm)**	Mean Difference (%)***	T Test Sig.
Supraglabellare	3.4	4.5	-1.1	-24.4	0.00*	4.3	5.5	-1.2	-21.8	0.00*
Glabella	5	4.9	0	0.0	0.94	5.8	5.9	-0.2	-3.4	0.45
Nasion	5.9	6.2	-0.3	-4.8	0.11	7.2	8.1	-1	-12.3	0.00*
Rhinion	1.7	1.7	0	0.0	0.61	1.9	2.3	-0.3	-13.0	0.01*
Mid-Philtrum	12.3	13.1	-0.7	-5.3	0.02*	15.1	15.7	-0.6	-3.8	0.16
Prosthion	9.5	10	-0.5	-5.0	0.17	12.4	13.3	-0.9	-6.8	0.02*
Infradentale	11.3	9.8	1.5	15.3	0.29	11.2	11.9	-0.8	-6.7	0.05*
Supramentale	10.8	11.9	-1.1	-9.2	0.00*	11.4	12.9	-1.5	-11.6	0.00*
Pogonion	9.4	9.8	-0.4	-4.1	0.42	10.8	11.4	-0.7	-6.1	0.21
Menton	6.9	7	-0.1	-1.4	0.87	8.6	9	-0.4	-4.4	0.38
Frontal Eminence R	3.5	4.1	-0.6	-14.6	0.00*	4.4	5	-0.6	-12.0	0.09
Frontal Eminence L	3.4	4.2	-0.8	-19.0	0.00*	4.5	4.9	-0.5	-10.2	0.21
Mid-supraorbital R	6.2	6.5	-0.3	-4.6	0.22	7.3	8.6	-1.3	-15.1	0.00*
Mid-supraorbital L	6.1	6.4	-0.2	-3.1	0.33	7.2	8.7	-1.6	-18.4	0.00*
Mid-infraorbital R	5	5.3	-0.3	-5.7	0.32	5.4	5.8	-0.4	-6.9	0.16
Mid-infraorbital L	4.8	5.5	-0.6	-10.9	0.03	5.4	5.9	-0.5	-8.5	0.07
Malar R	19.4	21.4	-2	-9.3	0.00*	20.3	22.8	-2.5	-11.0	0.00*
Malar L	18.7	21.7	-3	-13.8	0.00*	20.3	22.9	-2.6	-11.4	0.00*
Lateral Orbital R	9	9.1	-0.1	-1.1	0.76	7.5	8.3	-0.8	-9.6	0.01*
Lateral Orbital L	10.4	9.2	1.2	13.0	0.38	7.3	8.3	-1	-12.0	0.00*
Zygion R	7.4	7.8	-0.4	-5.1	0.27	8.1	9.2	-1.1	-12.0	0.01*
Zygion L	7.5	7.8	-0.3	-3.8	0.43	7.8	9.1	-1.4	-15.4	0.00*
Supraglenoid R	10	10.7	-0.7	-6.5	0.06	11.4	12.8	-1.4	-10.9	0.00*
Supraglenoid L	9.9	10.7	-0.8	-7.5	0.04*	11.1	12.8	-1.6	-12.5	0.00*
Gonion R	13.2	12.8	0.3	2.3	0.66	16.9	18.3	-1.4	-7.7	0.21
Gonion L	13.2	13	0.2	1.5	0.73	17.2	17.8	-0.6	-3.4	0.55
Ectomolare^2 R	26	27	-1	-3.7	0.11	28.2	30.3	-2.2	-7.3	0.01*
Ectomolare^2 L	26.3	27.2	-0.9	-3.3	0.17	28.2	30.2	-1.9	-6.3	0.01*
Occlusal Line R	20.1	20.3	-0.2	-1.0	0.73	22.8	24.6	-1.8	-7.3	0.01*
Occlusal Line L	20.4	20.4	0	0.0	0.93	23	24.3	-1.3	-5.3	0.07
Ectomolare$_2$ R	23.5	24.9	-1.3	-5.2	0.03*	25.1	28.2	-3.1	-11.0	0.00*
Ectomolare$_2$ L	24	24.8	-0.8	-3.2	0.18	25.5	28.4	-2.9	-10.2	0.00*

*$P < 0.05$

; * Differences calculated between the averages of SE sample compared to MW sample.

Discussion

FSTT is one of the most studied subjects in the FFR field [22]. It provides quantitative data for the technique [13], but it still has many uncovered issues that justify researchers' continuous interest. Studies have confirmed that FSTT measures obtained by using CBCT result in reliable and reproducible data [7,18,23,24].

Several studies have verified the presence of FSTT variances among different populations, suggesting the importance of using specific values for each one [8,10–12,25,26]. Some of these studies compared data of FSTT averages that were obtained with different measurement protocols with inevitable bias to those conclusions [13]. This may explain why there are other studies that contradict this hypothesis and do not support that specific FSTTs are needed for different populations for the FFR [27–29]. The FSTT measurement method is important to the analysis of the specificity among different studies [29]. Our study was able to compare regional FSTT using the same method from a previous study, eliminating the method variant.

The concordance results from the interexaminer ICC analysis showed the Beaini protocol [18] can provide reproducibility and that it is recommended for future research. The intra-examiner ICC analysis demonstrated high concordance on the measurements made by the main examiner, in accordance with other studies that utilized the CBCT with the same objective [18,30]. Nevertheless, it was a result of practical experience gained on previous training, which may be necessary and well demonstrated in future studies.

Compared to the previous research [18], that produced a FSTT chart from a Brazilian Southeastern sample, it was clear that the heterogeneity of the Brazilian population is marked by a diverse population affinity that could not be completely made clear [2]. Analyzing the average of the differences among the Southeastern and Midwestern samples, the negative variation shows a predominance of deeper FSTTs among the Midwestern sample when compared to the Southeastern sample.

Considering the differences related to sex in the samples of the Southeastern [18] and Midwestern regions (this paper), statistical differentiation was observed. In males, differences were more evident in 5 landmarks on the midline and 8 bilateral points. Quantitatively, that variance did not surpass 2.5 mm, except the $ectomolare_2$ with 3.1 mm. Proportionally, it varie between 10 to 15% in most cases with a few landmarks showing

higher values. Within females, studies indicate fewer divergent landmarks (3 on the midline and 8 bilateral) where only malar showed a difference above 2.5 mm (3.0 mm). These results reveal high compatibility among the samples, considering that errors lower than 2.5 mm, may result in high correspondence on a facial pool [31] and differences between 2.5 ≤ 2.9 mm have minimal practical impact [32]

Whether reconstructions should match local facial traits is not under debate, and the use of FSTT tables of a specific ancestry FSTT charts may provide better FFR. However, local changes should not be exclusively related to FSTT [33]. Eyes, lips, nose, and skin features could also be relevant in the differentiation of people from these 2 different areas in Brazil.

Investigating further into our results, they support that previous to the FFR, a careful anthropological evaluation of the skull regarding sex, age and ancestry variance remain necessary [6] because it provides important information to the forensic professional. However, the use of FSTT mean values divided by sex, age, and ancestry is no guarantee to lead to better recognition rates, and awareness of the complexity of soft tissue displacement caused by aging [34]. The assessment of the age variant was originally made to reduce sample distribution bias when compared to the Southeastern sample [18] but it presented interesting results. Our results

show that attention to this matter should not be neglected on reconstructed faces [27].

The same was planned to verify sex differences because the Southeastern research showed statistically significant changes in STT related to sex. In this research, in agreement with previous studies [18,35–37], the analysis among sexes revealed males tend to possess higher depths than females, excepting the lateral orbital landmark, in which it has been observed a mean difference of 0.8 mm.

However, such statistical difference may not be the only reason for sexual dimorphism features [9,36,38]. Landmarks showed FSTT averages variate thickness within a small quantitative interval (<6% average), leaving sex variance among the individuals to skull formation [38].

The variation between males and females was no greater than 3 mm in most points. Only 4 landmarks exhibited greater discrepancies and those were located at anatomical regions most influenced by the BMI [37], such as gonion (5.5 mm), ectomare^2 (3.3 mm), ectomloare$_2$ (3.6 mm) and occlusal line (4.4 mm). Hence, sexual dimorphism was not clearly evidenced in the variation of depths of soft tissue. Despite the statistical differences, the authors found that male and female individuals appear to share similar averages [38]. The human skull is the third best reference for determining

sex, after the pelvic bone and postcranial dimensions [39]. Therefore, the differences verified in this study indicate that sexual dimorphism is probably based, mainly, on the skull morphology.

Multiple facial morphological and physiological alterations act on the ageing process of the face. Its beginning and progression vary between individuals, sexes, and ancestries [40]. In this study, meaningful differences were observed in the FSTT among the age group, notably in females, being the landmarks located on the medium and inferior face. The FSTT averages of the landmarks mid-philtrum, prosthion and ectomolare^2 tend to decrease progressively with age, reproducing the morphological alteration patterns that occur in facial ageing, demonstrating a coherence among the data. With the advancing of age, the average face volume is permanently lost from both from superficial and deep fat tissue [41], and a redistribution of this volume occurs that causes slenderizing of the superior lip more than it does in the inferior one [42]. Bone remodeling in that region results in a decrease of the overlying soft tissue support area. This phenomenon, along with the effect of gravity, characterizes the average ageing of the face [34,43].

Conclusions

The measurements analyzed in this research, when compared to those collected in the Southeast region of Brazil, have statistically differed

in many landmarks. However, observing the averages in a specific FSTT chart, indicated that regional differences between the 2 samples are quantitatively small and they should have minimal influence on facial soft tissue thicknesses. Therefore, the use of specific FSTT charts is not necessary when performing FFR of individuals from Brazil's Southeast and Midwest.

References

1. Wilkinson C. Facial reconstruction – anatomical art or artistic anatomy? J Anat. 2010;216: 235–250. doi:10.1111/j.1469-7580.2009.01182.x

2. Wilkinson C. Computerized forensic facial reconstruction : A review of current systems. Forensic Sci Med Pathol. 2005;1: 173–177. doi:10.1385/FSMP:1:3:173

3. Wilkinson C. Facial identification of the dead. J Anat. 2008 [cited 29 Jun 2020]. doi:10.1111/j.1469-7580.2008.00972.x

4. Fernandes CMS, da Costa Serra M, da Silva JVL, Yoshito Noritomi P, de Sena Pereira FDA, Melani RFH. Tests of one Brazilian facial reconstruction method using three soft tissue depth sets and familiar assessors. Forensic Sci Int. 2012;214: 211.e1-211.e7. doi:10.1016/j.forsciint.2011.08.017

5. Stephan CN, Caple JM, Guyomarc'h P, Claes P. An overview of the latest developments in facial imaging. Forensic Sci Res. 2019;4: 10–28. doi:10.1080/20961790.2018.1519892

6. de Almeida NH, Michel-Crosato E, de Paiva LAS, Biazevic MGH. Facial soft tissue thickness in the Brazilian population: new reference data and anatomical landmarks. Forensic Sci Int. 2013;231: 404.e1–7. doi:10.1016/j.forsciint.2013.05.024

7. Fourie Z, Damstra J, Gerrits PO, Ren Y. Accuracy and reliability of facial soft tissue depth measurements using cone beam computer tomography. Forensic Sci Int. 2010;199: 9–14. doi:10.1016/j.forsciint.2010.02.018

8. Cavanagh D, Steyn M. Facial reconstruction: Soft tissue thickness values for South African black females. Forensic Sci Int. 2011;206: 215.e1-215.e7. doi:10.1016/j.forsciint.2011.01.009

9. Tedeschi-Oliveira SV, Melani RFH, de Almeida NH, de Paiva LAS. Facial soft tissue thickness of Brazilian adults. Forensic Sci Int. 2009;193: 127.e1-127.e7. doi:10.1016/j.forsciint.2009.09.002

10. Bulut O, Sipahioglu S, Hekimoglu B. Facial soft tissue thickness database for craniofacial reconstruction in the Turkish adult population. Forensic Sci Int. 2014;242: 44–61. doi:10.1016/j.forsciint.2014.06.012

11. Codinha S. Facial soft tissue thicknesses for the Portuguese adult population. Forensic Sci Int. 2009;184: 80.e1-80.e7. doi:10.1016/j.forsciint.2008.11.011

12. De Donno A, Sablone S, Lauretti C, Mele F, Martini A, Introna F, et al. Facial approximation: Soft tissue thickness values for Caucasian males using cone beam computer tomography. Leg Med. 2019;37: 49–53. doi:10.1016/j.legalmed.2019.01.002

13. Stephan CN, Simpson EK. Facial Soft Tissue Depths in Craniofacial Identification (Part I): An Analytical Review of the Published Adult Data*. J Forensic Sci. 2008 [cited 26 Jun 2020]. doi:10.1111/j.1556-4029.2008.00852.x

14. Parra FC, Amado RC, Lambertucci JR, Rocha J, Antunes CM, Pena SDJ. Color and genomic ancestry in Brazilians. Proc Natl Acad Sci. 2003;100: 177–182. doi:10.1073/pnas.0126614100

15. Gonzales PS, Machado CEP, Michel-Crosato E. Photoanthropometry of the Face in the Young White Brazilian Population. Braz Dent J. 2018;29: 619–623. doi:10.1590/0103-6440201802027

16. Jurda M, Urbanová P. Sex and ancestry assessment of Brazilian crania using semi-automatic mesh processing tools. Leg Med. 2016;23: 34–43. doi:10.1016/j.legalmed.2016.09.004

17. Cunha E, Lopez-Capp TT, Inojosa R, Marques SR, Moraes LOC, Liberti E, et al. The Brazilian identified human osteological collections. Forensic Sci Int. 2018;289: 449.e1-449.e6. doi:10.1016/j.forsciint.2018.05.040

18. Beaini TL, Miamoto P, Duailibi-Neto EF, Tedeschi-Oliveira SV, Chilvarquer I, Melani RFH. Facial soft tissue depth measurements in cone-beam computed tomography: A study of a Brazilian sample. Leg Med. 2021;50: 101866. doi:10.1016/j.legalmed.2021.101866

19. Dias PEM, Beaini TL, Melani RFH. Evaluation of osifix software with craniofacial anthropometric purposes. J Res Dent. 2013;1: 351. doi:10.19177/jrd.v1e42013351-367

20. Rhine JS, Campbell HR. Thickness of facial tissues in American blacks. J Forensic Sci. 1980;25: 847–858.

21. Haukoos JS. Advanced Statistics: Bootstrapping Confidence Intervals for Statistics with “Difficult” Distributions. Acad Emerg Med. 2005;12: 360–365. doi:10.1197/j.aem.2004.11.018

22. Stephan CN, Munn L, Caple J. Facial soft tissue thicknesses: Noise, signal, and P. Forensic Sci Int. 2015;257: 114–122. doi:10.1016/j.forsciint.2015.07.051

23. Hwang H-S, Kim K, Moon D-N, Kim J-H, Wilkinson C. Reproducibility of Facial Soft Tissue Thicknesses for Craniofacial Reconstruction Using Cone-Beam CT Images: FACIAL SOFT TISSUE THICKNESS USING CBCT IMAGES. J Forensic Sci. 2012;57: 443–448. doi:10.1111/j.1556-4029.2011.02007.x

24. Hwang H-S, Choe S-Y, Hwang J-S, Moon D-N, Hou Y, Lee W-J, et al. Reproducibility of Facial Soft Tissue Thickness Measurements Using Cone-Beam CT Images According to the Measurement Methods. J Forensic Sci. 2015;60: 957–965. doi:10.1111/1556-4029.12766

25. Phillips VM, Smuts NA. Facial reconstruction: utilization of computerized tomography to measure facial tissue thickness in a mixed racial population. Forensic Sci Int. 1996;83: 51–59. doi:10.1016/0379-0738(96)02010-5

26. Baillie LJ, Mirijali SA, Niven BE, Blyth P, Dias GJ. Ancestry and BMI Influences on Facial Soft Tissue Depths for A Cohort of Chinese and Caucasoid Women in Dunedin, New Zealand. J Forensic Sci. 2015;60: 1146–1154. doi:10.1111/1556-4029.12799

27. Lee W-J, Wilkinson CM, Hwang H-S, Lee S-M. Correlation Between Average Tissue Depth Data and Quantitative Accuracy of Forensic Craniofacial Reconstructions Measured by Geometric Surface Comparison Method. J Forensic Sci. 2015;60: 572–580. doi:10.1111/1556-4029.12726

28. Thiemann N, Keil V, Roy U. In vivo facial soft tissue depths of a modern adult population from Germany. Int J Legal Med. 2017;131: 1455–1488. doi:10.1007/s00414-017-1581-y

29. Guyomarc'h P, Santos F, Dutailly B, Coqueugniot H. Facial soft tissue depths in French adults: variability, specificity and estimation. Forensic Sci Int. 2013;231: 411.e1–10. doi:10.1016/j.forsciint.2013.04.007

30. Baccarin LS. Análise da relação entre as espessuras médias de tecidos moles da face de crianças e adultos para reconstrução facial forense. Doutorado, Universidade de São Paulo. 2019.

31. Stephan CN. Facial Approximation-From Facial Reconstruction Synonym to Face Prediction Paradigm. J Forensic Sci. 2015;60: 566–571. doi:10.1111/1556-4029.12732

32. Briers N, Steyn M. Re-assessment of South African juvenile facial soft tissue thickness data for craniofacial approximation: A comparative analysis using central tendency statistics. Forensic Sci Int. 2018;291: 280.e1-280.e13. doi:10.1016/j.forsciint.2018.08.008

33. Wilkinson C, Neave R, Smith D. How important to facial reconstruction are the correct ethnic group tissue depths? Proceedings of the 10th Conference of the International Association of Craniofacial Identification. Bari, Italy: University of Bari; 2002. pp. 111–21.

34. Buchanan DR, Wulc AE. Contemporary Thoughts on Lower Eyelid/Midface Aging. Clin Plast Surg. 2015;42: 1–15. doi:10.1016/j.cps.2014.09.003

35. Farias Gomes A, Moreira DD, Zanon MF, Groppo FC, Haiter-Neto F, Freitas DQ. Soft tissue thickness in Brazilian adults of different skeletal classes and facial types: A cone beam CT – Study. Leg Med. 2020;47: 101743. doi:10.1016/j.legalmed.2020.101743

36. Dong Y, Huang L, Feng Z, Bai S, Wu G, Zhao Y. Influence of sex and body mass index on facial soft tissue thickness measurements of the northern Chinese adult population. Forensic Sci Int. 2012;222: 396.e1-396.e7. doi:10.1016/j.forsciint.2012.06.004

37. De Greef S, Vandermeulen D, Claes P, Suetens P, Willems G. The influence of sex, age and body mass index on facial soft tissue depths. Forensic Sci Med Pathol. 2009;5: 60–65. doi:10.1007/s12024-009-9085-9

38. Stephan CN, Norris RM, Henneberg M. Does sexual dimorphism in facial soft tissue depths justify sex distinction in craniofacial identification? J Forensic Sci. 2005;50: 513–518.

39. Nikita E, Michopoulou E. A quantitative approach for sex estimation based on cranial morphology. Am J Phys Anthropol. 2018;165: 507–517. doi:10.1002/ajpa.23376

40. Cotofana S, Gotkin RH, Ascher B, Morozov SP, Gombolevsky VA, Laipan AS, et al. Calvarial Volume Loss and Facial Aging: A Computed Tomographic (CT)-Based Study. Aesthet Surg J. 2018;38: 1043–1051. doi:10.1093/asj/sjy096

41. Boehm LM, Morgan A, Hettinger P, Matloub HS. Facial Aging: A Quantitative Analysis of Midface Volume Changes over 11 Years. Plast Reconstr Surg. 2021;147: 319–327. doi:10.1097/PRS.0000000000007518

42. Ko AC, Korn BS, Kikkawa DO. The aging face. Surv Ophthalmol. 2017;62: 190–202. doi:10.1016/j.survophthal.2016.09.002

43. Mendelson B, Wong C-H. Changes in the Facial Skeleton With Aging: Implications and Clinical Applications in Facial Rejuvenation. Aesthetic Plast Surg. 2012;36: 753–760. doi:10.1007/s00266-012-9904-3

The authors would like to thank the reviewers for their valuable comments and excellent suggestions to improve the quality of the manuscript entitled "*Facial soft tissue thickness in forensic facial reconstruction: influence of regional differences in Brazil"* (PONE-D-22-02392).

The authors have revised all points brought by the reviewers. We sincerely hope that the information presented in this manuscript provides some answers and opens novel questions to advance future research.

Reviewer #1

(Reviewer 1) Language editing needed – I indicated these by yellow highlighting in the attached document as they were too much to correct.

Action: The authors agree with the reviewer's point and as requested, a professional revision was performed by papercheck (DocID: 853143) to improve the fluidity and better understanding of the text.

(Reviewer 1) Overall, I think the research is sound, although not very innovative in the sense that it repeats what others have said before. We will need a better discussion of the results, and based on this and other studies clear guidance on the way forward with regard to the use of STT tables. What is the most important – age? Sex? Population?

Action: The authors would like to thank for the commentary, and we revised the discussion to meet the purpose of offering a clear comprehension and guidance on the way forward regarding the use of FSTT tables.

(Reviewer 1) Title: Add "in Brazil" at the end

Action: Added "in Brazil" at the end. Additionally, the authors changed the title to "*Facial soft tissue thickness in forensic facial reconstruction: influence of regional differences in Brazil"*, emphasizing the population, which is the focus of this article.

Abstract:

(Reviewer 1) Please provide the sample sizes.

Action: The request was attended at line 41: *"A specific protocol for cone beam computed tomography was used to standardize measurement, and it was applied in a sample of 101 exams."*

Introduction:

(Reviewer 1) The debate about the use of population-specific STT is not fully explored. This needs more detail.

Action: The authors agree with the reviewer's point and, as requested, we delineate the use of population-specific FSTT in Lines 82 to 98. The Introduction was enhanced with description of populational characteristics in Brazil. The discussion section was reformulated to highlight this subject that is our main objective.

(Reviewer 1) We need to know more as to why the two populations are expected to be different – i.e., the background of the samples. The Intro needs to better reflect current debates around the issues raised.

Action: Thank you to point that out, as requested, we clarify this issue in Lines 99 and 121 of the Introduction section, also some parts of the discussion brings the debate to our results.

Material and Methods

(Reviewer 1) We need info on the age and sex distribution of the sample (found this later in Results – should be in M and M). Sample sizes in each sex-age cohort quite small (as few as n = 13). This is a shortcoming that should be discussed later on. We also need an indication of the sample sizes of the comparative sample.

Action: As requested by the reviewer, the authors moved the Distribution of the sample to M and M (Line 128). Sample sizes in each sex-age cohort are limited to Radiology Institute's availability according to the inclusion criteria (unfortunately there is little availability of tomography exams that have compatible FOV- filed of view). Furthermore, in lines 164 to 167, sample sizes of the comparative sample were added.

(Reviewer 1) 'I don't know the software used – cant comment on it'

Action: The authors provided this information, in lines 160 to 162: *"Horos software is an open access version of OsiriX (DICOM viewer software) and has demonstrated to be a good alternative for craniofacial anthropological analyses, both in hard and soft tissues (Dias et al., 2013)."* Also, we would like to point out that Horos was used in the original research due to the possibility of repositioning the volume prior to the measurements. This is an important step as it eliminates patient positioning issues and standardize the measurements, increasing the reproducibility.

(Reviewer 1) I assume the individuals were in a sitting position when scanned? – this needs better discussion also with regards to best practice and current guidelines.

Action: The authors agree with the reviewer, and as requested, we explain this issue in Lines 138 to 141: *"CBCT scans allow image acquisitions with the individual in a sitting or standing position. Despite containing stabilizing devices for the head, such as the cephalostat and the chin support, these were not used in our sample, which avoided the effects of compression of facial soft tissues."*

(Reviewer 1) Inter- and intra-observer repeatability assessment needs better explanation

Action: The authors agree with the reviewer's point and as requested, we explain this issue in lines 194 to 199: *"The evaluation and adjustment of inter and intra-examiners was achieved by testing the 32 landmarks in 15 exams (15% of the sample) and testing the intraclass correlation coefficient (ICC) for each point. Regarding intra-examiner*

assessment, the main examiner measured the 15 exams twice, considering 1 week time interval between the measurements."

(Reviewer 1) Comparison between the two regions – are the people from the two regions supposed to be different? Why would we expect them to be different? This needs explanation.

Action: The authors agree with the reviewer's point and as requested, they explain this issue in the Introduction section in lines 98 a 117.

Results

(Reviewer 1) The inter- and intra-observer results are too uninformative, we need the actual results. Inter-observer above 95% is really unusual.

Action: The authors added a chart to supplement the information requested, line 224: *" Fig 4 presents the individual ICC records."*

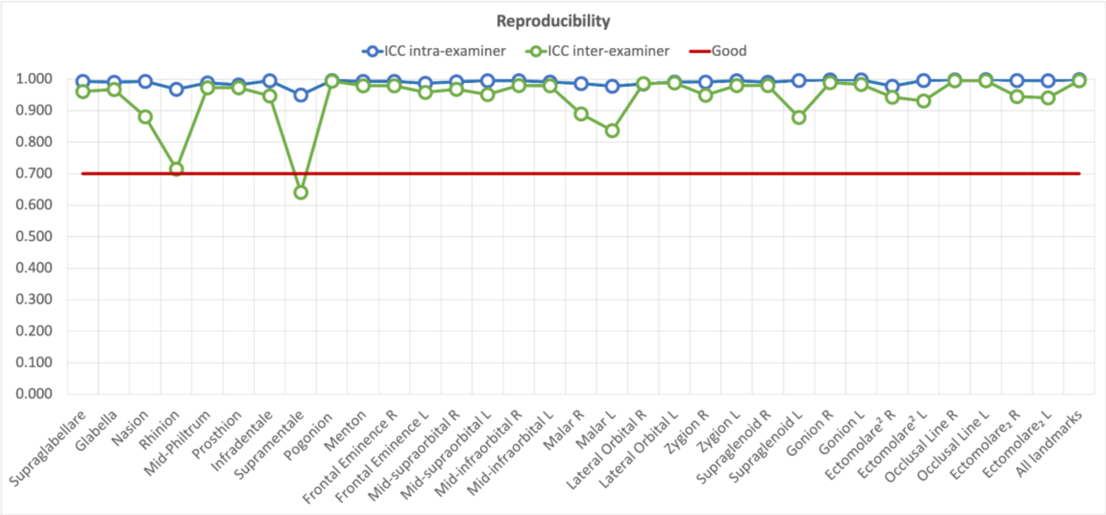

(Reviewer 1) Table with sample sizes should be in the M and M. In the end, the sample size per sex-age group is really small (with as few as 13 individuals)

Action: Distribution of the sample transferred to M and M. Line 128

(Reviewer 1) Differences between the two compared groups should be discussed in more detail – how many measurements differed significantly? It seems there were quite a few. When does one decide that these differences are enough to use population-specific data? How much difference would this make in an actual reconstruction? In the literature it was suggested that one should look not so much at the absolute difference in mm, but rather in % of the actual difference (e.g., a 2 mm difference in a measurement of 10 mm may not make much of a difference in a reconstruction, but 2 mm when the average is 5 mm will be more pronounced)….Think it was a paper by Briers? This warrants better Discussion (in the Discussion section).

Action: We agree with the reviewer's comment in the sense that proportional difference may reflect better the actual differences related to FSTT, Thus, we added a column in table 3 and 4 to observe how much difference (in %) it would represent. Subsequently, we added this point to the discussion in lines 304 to 306 and 310 to 311.

Discussion

(Reviewer 1) Changes with age cannot be ascribed to bone losses only – the soft tissues as well as the hard tissues undergo changes. Changes with age needs a more thorough discussion with reference to the literature– seems that in this study this was more important than differences between populations from different regions – would you advise that separate tables are used for older and younger groups? But not for different populations?

Action: The reviewer is right in this matter. Even though some differences are evident between age ranges in the research, it does not fully explain the changes related to the aging process.

(Reviewer 1) **Miscegenation** is a strong word to use, with negative connotations. Please rephrase.

Action: Thank you for saying that. Sometimes language differences may give the wrong interpretation of the chosen words. It was rephrased as requested.

(Reviewer 1) It is stated that the regional differences have an insignificant impact on facial soft tissue thicknesses and consequently on FFR result. This last part – the actual result – has not been tested in this study and this statement can thus not be substantiated. It may be suggested, but was not proven by this study.

Action: The authors agree with the reviewer's recommendation, and we rephrased the conclusion.

Overall, we need to be advised on the way forward, based on the results from this (and other) studies. The last sentence of the conclusion seems to contradict all of what had gone before – do we still need to take population, sex etc into account or not?

Action: Thank you for the observation, we rephrased the conclusion.

Reviewer #2

Overall, the manuscript is written well and logically laid out. The authors have provided details of demonstrated rigour with regards to the experimental design and subsequent analysis through inter and intra observer error, tests for normality, and bootstrapping where appropriate. A narrative is built up throughout the paper by describing the methodology in a chronological fashion, which I found to be well considered. All supporting data appears to have been made fully available.

The consideration of the level of specificity and granularity required with FSTTs is an important one, and it was interesting to see that the differences at regional level for Brazil were insufficient to justify multiple FSTT datasets.

I've noted a few suggestions below. Additionally, I have made some suggested edits for the English, as in some cases it was a little unclear what you were intending to

say. These are small suggestions for the authors to consider, but I would encourage the inclusion of further clarification around some of the points raised:

General comments:

You cover the literature in terms of the 'success' of population specific datasets in FFR, and that incongruent FSTTs still yield a recognisable face, but that congruent population specific datasets might result in a more 'accurate' FFR, although differences in protocols might suggest insufficient evidence for this. I wonder if you could provide some more background on the importance of FSTTs in the recognition process. I.e. FSTTs mainly cover the contours of the face, not the estimation of facial features. Literature suggests facial features and their configuration are more important for familiar face recognition. Furthermore, given the variation of FSTTs in the facial contour due to BMI and ageing, we have a greater tolerance for inaccuracies in these areas. I think it would be useful for you to provide some background on this and to discuss the relative importance of FSTT datasets in the Facial reconstruction and subsequent recognition process. You touch on this briefly in the discussion, but more is needed.

Action: The authors would like to thank for the observation. There are few articles that debates this issue. However, the authors considered what is in the literature, references such as Stephan and Simpson and Briers, whose studies demonstrated that differences under 3 mm have little practical impact in FFR. Besides, it is necessary to consider that in FFR the result is not only associated to FSTT, but also to craniofacial biotype.

Lines 273 – 283: Can you provide information of the distribution of samples in the above 41yrs age bracket? i.e. the range? As you mention, age-related changes to the soft tissues change in 'type' as age increases. For example, someone in their 40s may have superficial age related changes, caused by a change to the dermis. But those in their 60s and 70s are more likely to also exhibit more extreme volume/morphological changes, such as ptosis etc. which would have a bigger impact on the position of FSTTs. Given this, it would be useful to know the age range for that bracket, and if there was a

wide range, why the sample was collapsed into one group. Especially given that a large portion of this manuscript discusses age-related changes.

Action: The authors agree with the points mentioned about age related changes. However, despite the differences between someone in their 40s and those in their 60s (our sample range), we collapsed into one group. The literature demonstrates that, since 40s, the hormonal impact on muscle mass and skin flaccidity is gradual and significant, even though these changes occur in distinct levels.

Do you anticipate any plans for future research in this area? Given that you observe morphological variation in faces between regions of Brazil, enough to warrant your investigation into FSTTs, might it be worth investigating parameters for feature estimation between these regions? It would be great to see this research carried out.

Action: Thank you for the question. Yes, this research is part of a major project that will gradually investigate facial features of the Brazilian population. Due to the difficulty to access CBCT exams, this project has been divided by regions. We are glad to know you share the same interest.

Formatting edits:

Perhaps increase the size of the annotation text on figures 2 and 3? If I download the figures, I can see it more clearly when zoomed in, but they are barely readable when in article format.

Action: Indeed, it needed reformatting. The annotation text on figures 2 and 3 were resized.

The Ectomolare could be better differentiated rather than using superscript and subscript 2. Whilst it represents upper and lower, I think it's quite hard to see visually. Maybe 1 and 2 is better?

Action: Caple and Stephan (2016) are the reference article for the definition of the craniometric landmarks, and they represent the upper ectomolare using superscript 2, and the lower ectomolare using subscript 2.

Grammatical edits:

Action: A professional revision was performed by papercheck (DocID: 853143) to improve the fluidity and better understanding of the text.
