# Supplementary material for: Facial soft tissue thickness in forensic facial reconstruction: Impact of regional differences in Brazil
Source: PLoS One. 2022 Jul 15;17(7):e0270980. doi: 10.1371/journal.pone.0270980 (PMC9286276; doi:10.1371/journal.pone.0270980)
Supplement: Attachment — Submitted filename: Response to Reviewers.docx [file pone.0270980.s003.docx]

The authors would like to thank the reviewers for their valuable comments and excellent suggestions to improve the quality of the manuscript entitled “*Facial soft tissue thickness in forensic facial reconstruction: influence of regional differences in Brazil”* (PONE-D-22-02392).

The authors have revised all points brought by the reviewers. We sincerely hope that the information presented in this manuscript provides some answers and opens novel questions to advance future research.

**Reviewer #1**

**(Reviewer 1)** Language editing needed – I indicated these by yellow highlighting in the attached document as they were too much to correct.

**Action:** The authors agree with the reviewer’s point and as requested, a professional revision was performed by papercheck (DocID: 853143) to improve the fluidity and better understanding of the text.

**(Reviewer 1)** Overall, I think the research is sound, although not very innovative in the sense that it repeats what others have said before. We will need a better discussion of the results, and based on this and other studies clear guidance on the way forward with regard to the use of STT tables. What is the most important – age? Sex? Population?

**Action:** The authors would like to thank for the commentary, and we revised the discussion to meet the purpose of offering a clear comprehension and guidance on the way forward regarding the use of FSTT tables.

**(Reviewer 1)** Title: Add “in Brazil” at the end

**Action:** Added "in Brazil" at the end. Additionally, the authors changed the title to “*Facial soft tissue thickness in forensic facial reconstruction: influence of regional differences in Brazil”,* emphasizing the population, which is the focus of this article.

**Abstract:**

**(Reviewer 1)** Please provide the sample sizes.

**Action:** The request was attended at line 41: *“A specific protocol for cone beam computed tomography was used to standardize measurement, and it was applied in a sample of 101 exams.”*

**Introduction**:

**(Reviewer 1)** The debate about the use of population-specific STT is not fully explored. This needs more detail.

**Action:** The authors agree with the reviewer’s point and, as requested, we delineate the use of population-specific FSTT in Lines 82 to 98. The Introduction was enhanced with description of populational characteristics in Brazil. The discussion section was reformulated to highlight this subject that is our main objective.

**(Reviewer 1)** We need to know more as to why the two populations are expected to be different – i.e., the background of the samples. The Intro needs to better reflect current debates around the issues raised.

**Action:** Thank you to point that out, as requested, we clarify this issue in Lines 99 and 121 of the Introduction section, also some parts of the discussion brings the debate to our results.

**Material and Methods**

**(Reviewer 1)** We need info on the age and sex distribution of the sample (found this later in Results – should be in M and M). Sample sizes in each sex-age cohort quite small (as few as n = 13). This is a shortcoming that should be discussed later on. We also need an indication of the sample sizes of the comparative sample.

**Action:** As requested by the reviewer, the authors moved the Distribution of the sample to M and M (Line 128). Sample sizes in each sex-age cohort are limited to Radiology Institute’s availability according to the inclusion criteria (unfortunately there is little availability of tomography exams that have compatible FOV- filed of view). Furthermore, in lines 164 to 167, sample sizes of the comparative sample were added.

**(Reviewer 1)** ‘I don’t know the software used – cant comment on it’

**Action:** The authors provided this information, in lines 160 to 162: *"Horos software is an open access version of OsiriX (DICOM viewer software) and has demonstrated to be a good alternative for craniofacial anthropological analyses, both in hard and soft tissues (Dias et al., 2013)."* Also, we would like to point out that Horos was used in the original research due to the possibility of repositioning the volume prior to the measurements. This is an important step as it eliminates patient positioning issues and standardize the measurements, increasing the reproducibility.

**(Reviewer 1)** I assume the individuals were in a sitting position when scanned? – this needs better discussion also with regards to best practice and current guidelines.

**Action:** The authors agree with the reviewer, and as requested, we explain this issue in Lines 138 to 141: *“CBCT scans allow image acquisitions with the individual in a sitting or standing position. Despite containing stabilizing devices for the head, such as the cephalostat and the chin support, these were not used in our sample, which avoided the effects of compression of facial soft tissues.”*

**(Reviewer 1)** Inter- and intra-observer repeatability assessment needs better explanation

**Action:** The authors agree with the reviewer’s point and as requested, we explain this issue in lines 194 to 199: *"The evaluation and adjustment of inter and intra-examiners was achieved by testing the 32 landmarks in 15 exams (15% of the sample) and testing the intraclass correlation coefficient (ICC) for each point. Regarding intra-examiner assessment, the main examiner measured the 15 exams twice, considering 1 week time interval between the measurements."*

**(Reviewer 1)** Comparison between the two regions – are the people from the two regions supposed to be different? Why would we expect them to be different? This needs explanation.

**Action:** The authors agree with the reviewer’s point and as requested, they explain this issue in the Introduction section in lines 98 a 117.

**Results**

**(Reviewer 1)** The inter- and intra-observer results are too uninformative, we need the actual results. Inter-observer above 95% is really unusual.

**Action:** The authors added a chart to supplement the information requested, line 224: *“ Fig 4 presents the individual ICC records.”*

**(Reviewer 1)** Table with sample sizes should be in the M and M. In the end, the sample size per sex-age group is really small (with as few as 13 individuals)

**Action:** Distribution of the sample transferred to M and M. Line 128

**(Reviewer 1)** Differences between the two compared groups should be discussed in more detail – how many measurements differed significantly? It seems there were quite a few. When does one decide that these differences are enough to use population-specific data? How much difference would this make in an actual reconstruction? In the literature it was suggested that one should look not so much at the absolute difference in mm, but rather in % of the actual difference (e.g., a 2 mm difference in a measurement of 10 mm may not make much of a difference in a reconstruction, but 2 mm when the average is 5 mm will be more pronounced)….Think it was a paper by Briers? This warrants better Discussion (in the Discussion section).

**Action:** We agree with the reviewer’s comment in the sense that proportional difference may reflect better the actual differences related to FSTT, Thus, we added a column in table 3 and 4 to observe how much difference (in %) it would represent. Subsequently, we added this point to the discussion in lines 304 to 306 and 310 to 311.

**Discussion**

**(Reviewer 1)** Changes with age cannot be ascribed to bone losses only – the soft tissues as well as the hard tissues undergo changes. Changes with age needs a more thorough discussion with reference to the literature– seems that in this study this was more important than differences between populations from different regions – would you advise that separate tables are used for older and younger groups? But not for different populations?

**Action:** The reviewer is right in this matter. Even though some differences are evident between age ranges in the research, it does not fully explain the changes related to the aging process.

**(Reviewer 1) Miscegenation** is a strong word to use, with negative connotations. Please rephrase.

**Action:** Thank you for saying that. Sometimes language differences may give the wrong interpretation of the chosen words. It was rephrased as requested.

**(Reviewer 1)** It is stated that the regional differences have an insignificant impact on facial soft tissue thicknesses and consequently on FFR result. This last part – the actual result – has not been tested in this study and this statement can thus not be substantiated. It may be suggested, but was not proven by this study.

**Action:** The authors agree with the reviewer’s recommendation, and we rephrased the conclusion.

Overall, we need to be advised on the way forward, based on the results from this (and other) studies. The last sentence of the conclusion seems to contradict all of what had gone before – do we still need to take population, sex etc into account or not?

**Action:** Thank you for the observation, we rephrased the conclusion.

**Reviewer #2**

Overall, the manuscript is written well and logically laid out. The authors have provided details of demonstrated rigour with regards to the experimental design and subsequent analysis through inter and intra observer error, tests for normality, and bootstrapping where appropriate. A narrative is built up throughout the paper by describing the methodology in a chronological fashion, which I found to be well considered. All supporting data appears to have been made fully available.

The consideration of the level of specificity and granularity required with FSTTs is an important one, and it was interesting to see that the differences at regional level for Brazil were insufficient to justify multiple FSTT datasets.

I’ve noted a few suggestions below. Additionally, I have made some suggested edits for the English, as in some cases it was a little unclear what you were intending to say. These are small suggestions for the authors to consider, but I would encourage the inclusion of further clarification around some of the points raised:

**General comments:**

You cover the literature in terms of the ‘success’ of population specific datasets in FFR, and that incongruent FSTTs still yield a recognisable face, but that congruent population specific datasets might result in a more ‘accurate’ FFR, although differences in protocols might suggest insufficient evidence for this. I wonder if you could provide some more background on the importance of FSTTs in the recognition process. I.e. FSTTs mainly cover the contours of the face, not the estimation of facial features. Literature suggests facial features and their configuration are more important for familiar face recognition. Furthermore, given the variation of FSTTs in the facial contour due to BMI and ageing, we have a greater tolerance for inaccuracies in these areas. I think it would be useful for you to provide some background on this and to discuss the relative importance of FSTT datasets in the Facial reconstruction and subsequent recognition process. You touch on this briefly in the discussion, but more is needed.

**Action:** The authors would like to thank for the observation. There are few articles that debates this issue. However, the authors considered what is in the literature, references such as Stephan and Simpson and Briers, whose studies demonstrated that differences under 3 mm have little practical impact in FFR. Besides, it is necessary to consider that in FFR the result is not only associated to FSTT, but also to craniofacial biotype.

Lines 273 – 283: Can you provide information of the distribution of samples in the above 41yrs age bracket? i.e. the range? As you mention, age-related changes to the soft tissues change in ‘type’ as age increases. For example, someone in their 40s may have superficial age related changes, caused by a change to the dermis. But those in their 60s and 70s are more likely to also exhibit more extreme volume/morphological changes, such as ptosis etc. which would have a bigger impact on the position of FSTTs. Given this, it would be useful to know the age range for that bracket, and if there was a wide range, why the sample was collapsed into one group. Especially given that a large portion of this manuscript discusses age-related changes.

**Action:** The authors agree with the points mentioned about age related changes. However, despite the differences between someone in their 40s and those in their 60s (our sample range), we collapsed into one group. The literature demonstrates that, since 40s, the hormonal impact on muscle mass and skin flaccidity is gradual and significant, even though these changes occur in distinct levels.

Do you anticipate any plans for future research in this area? Given that you observe morphological variation in faces between regions of Brazil, enough to warrant your investigation into FSTTs, might it be worth investigating parameters for feature estimation between these regions? It would be great to see this research carried out.

**Action:** Thank you for the question. Yes, this research is part of a major project that will gradually investigate facial features of the Brazilian population. Due to the difficulty to access CBCT exams, this project has been divided by regions. We are glad to know you share the same interest.

**Formatting edits:**

Perhaps increase the size of the annotation text on figures 2 and 3? If I download the figures, I can see it more clearly when zoomed in, but they are barely readable when in article format.

**Action:** Indeed, it needed reformatting. The annotation text on figures 2 and 3 were resized.

The Ectomolare could be better differentiated rather than using superscript and subscript 2. Whilst it represents upper and lower, I think it’s quite hard to see visually. Maybe 1 and 2 is better?

**Action:** Caple and Stephan (2016) are the reference article for the definition of the craniometric landmarks, and they represent the upper ectomolare using superscript 2, and the lower ectomolare using subscript 2.

Grammatical edits:

**Action:** A professional revision was performed by papercheck (DocID: 853143) to improve the fluidity and better understanding of the text.
