# Supplementary material for: Facial soft tissue thickness in forensic facial reconstruction: Impact of regional differences in Brazil
Source: PLoS One. 2022 Jul 15;17(7):e0270980. doi: 10.1371/journal.pone.0270980 (PMC9286276; doi:10.1371/journal.pone.0270980)
Supplement: Attachment — Submitted filename: Response to Reviewers.docx [file pone.0270980.s006.docx]

The authors would like to thank the reviewers and the Editor for their valuable comments that definitely improved the quality of the manuscript entitled “*Facial soft tissue thickness in forensic facial reconstruction: Impact of regional differences in Brazil”* (PONE-D-22-02392R1).

**Additional Editor Comments:**

All corrections have been made except one:
Line 27 should read: 'appearance of a face over a skull, in order to lead to recognition of that individual, making possible the application of primary identification methods'.

**Action:** The authors would like to apologize for this lapse, and as requested by the Editor, the authors revised the paper and the correction in Line 27 (highlighted) was made.
